# Unsaturated fatty acid perturbation combats emerging triazole antifungal resistance in the human fungal pathogen *Aspergillus fumigatus*

**DOI:** 10.1128/mbio.01166-24

**Published:** 2024-06-27

**Authors:** Cecilia Gutierrez-Perez, Charles Puerner, Jane T. Jones, Sandeep Vellanki, Elisa M. Vesely, Mark A. Xatse, Andre F. C. Viera, Carissa P. Olsen, Keren O. Attiku, Steven Cardinale, Steven M. Kwasny, Narendran G-Dayanandan, Timothy J. Opperman, Robert A. Cramer

**Affiliations:** 1 Microbiology and Immunology Department, Geisel School of Medicine, Dartmouth College, Hanover, New Hampshire, USA; 2 Department of Chemistry and Biochemistry, Worcester Polytechnic Institute, Worcester, Massachusetts, USA; 3 Microbiotix Inc., Worcester, Massachusetts, USA; Duke University, Durham, North Carolina, USA

**Keywords:** *Aspergillus fumigatus*, antifungal agents, sterol regulatory element binding protein, fatty acids, antifungal resistance

## Abstract

Contemporary antifungal therapies utilized to treat filamentous fungal infections are inhibited by intrinsic and emerging drug resistance. Consequently, there is an urgent need to develop novel antifungal compounds that are effective against drug-resistant filamentous fungi. Here, we utilized an *Aspergillus fumigatus* cell-based high-throughput screen to identify small molecules with antifungal activity that also potentiated triazole activity. The screen identified 16 hits with promising activity against *A. fumigatus*. A nonspirocyclic piperidine, herein named MBX-7591, exhibited synergy with triazole antifungal drugs and activity against pan-azole-resistant *A. fumigatus* isolates. MBX-7591 has additional potent activity against *Rhizopus* species and CO_2_-dependent activity against *Cryptococcus neoformans*. Chemical, genetic, and biochemical mode of action analyses revealed that MBX-7591 increases cell membrane saturation by decreasing oleic acid content. MBX-7591 has low toxicity *in vivo* and shows good efficacy in decreasing fungal burden in a murine model of invasive pulmonary aspergillosis. Taken together, our results suggest MBX-7591 is a promising hit with a novel mode of action for further antifungal drug development to combat the rising incidence of triazole-resistant filamentous fungal infections.

**IMPORTANCE:**

The incidence of infections caused by fungi continues to increase with advances in medical therapies. Unfortunately, antifungal drug development has not kept pace with the incidence and importance of fungal infections, with only three major classes of antifungal drugs currently available for use in the clinic. Filamentous fungi, also called molds, are particularly recalcitrant to contemporary antifungal therapies. Here, a recently developed *Aspergillus fumigatus* cell reporter strain was utilized to conduct a high-throughput screen to identify small molecules with antifungal activity. An emphasis was placed on small molecules that potentiated the activity of contemporary triazole antifungals and led to the discovery of MBX-7591. MBX-7591 potentiates triazole activity against drug-resistant molds such as *A. fumigatus* and has activity against Mucorales fungi. MBX-7591’s mode of action involves inhibiting the conversion of saturated to unsaturated fatty acids, thereby impacting fungal membrane integrity. MBX-7591 is a novel small molecule with antifungal activity poised for lead development.

## INTRODUCTION

Invasive fungal infections are difficult to diagnose and treat. These difficulties in diagnosis and treatment are reflected in the estimated ~1.5 million people per year who succumb to fungal infections, which is about the same as the number of people worldwide with tuberculosis and three times as many affected by malaria (1, 2). The most common causal agent of invasive filamentous fungal human infections is *Aspergillus fumigatus*. Over 2 million people develop invasive aspergillosis (IA) from which ~85% of patients succumb to the infection (2). Currently, there are only three major antifungal drug classes (triazoles, polyenes, and echinocandins) used to treat IA and most other human fungal infections (3, 4). The triazole class of antifungals inhibits ergosterol biosynthesis through targeted inhibition of the cytochrome P450 14-α sterol demethylase Cyp51 enzymes (also called Erg11p in yeast). Voriconazole (VCZ) remains the first-line therapy for IA cases, though newer triazoles such as isavuconazole are increasingly utilized (5, 6). Triazole use and efficacy face several challenges including, but not limited to, the emergence of triazole-resistant strains in previously susceptible species such as *A. fumigatus* (7–10). In some medical centers, up to 19% of *A. fumigatus* isolates are triazole resistant (11). Triazole-resistant isolates impact clinical outcomes as patients with a triazole-resistant fungal infection have a 20%–30% increase in mortality rate (11). Overall, it is estimated that contemporary antifungals fail in about 50% of patients with invasive filamentous fungal infections (6). Taken together, these observations are indicative of an urgent need to develop new antifungal agents with novel mechanisms of action (12, 13).

In addition to *cyp51A* allelic variants leading to triazole drug resistance, additional biological factors contribute to poor treatment outcomes, including biofilm formation and physiological adaptation to hypoxia in host tissues, which are regulated by a genetic network controlled by the *A. fumigatus* sterol-regulatory element binding protein transcription factor, SrbA (14–18). SrbA is a direct transcriptional regulator of the *cyp51A* gene encoding the lanosterol demethylase target of the triazoles (16–18). Many triazole-resistant strains contain a 34-base pair tandem repeat of the SrbA DNA binding motif that increases *cyp51A* expression levels (8, 18). Moreover, SrbA is required for the formation of drug-resistant biofilms, intrinsic resistance to fluconazole, and for virulence (15, 19). Loss of *srbA* dramatically increases susceptibility to triazoles, even in isolates with acquired triazole drug resistance (20). These data suggest that inhibition of SrbA activity and/or key members of the SrbA genetic network could overcome many of the challenges that reduce triazole drug efficacy.

To address the need for novel compounds with antifungal activity against *A. fumigatus* and other filamentous fungi, we designed a high-throughput cell-based screen to identify small molecules that inhibit the SrbA regulatory pathway or the biochemical pathways that are regulated by SrbA. From this screen, we identified the small molecule MBX-7591 that has micromolar-level antifungal activity against *A. fumigatus* and other human pathogenic fungi. Similar to the loss of *srbA,* MBX-7591 has synergy with triazole antifungal drugs *in vitro* against laboratory reference strains and triazole-resistant clinical and environmental isolates. Biochemical and chemical genetics data suggest that MBX-7591 directly or indirectly inhibits the essential stearoyl-CoA 9-desaturase, SdeA, which is directly regulated by SrbA and additional co-regulatory transcription factors HapX and AtrR (18, 21–23). Significantly, MBX-7591 was effective in decreasing fungal burden in a murine model of invasive pulmonary aspergillosis. Taken together, our results suggest that MBX-7591 is a promising new small molecule with a novel antifungal target that can be optimized to combat the emergence of triazole drug resistance in filamentous fungi.

## RESULTS

### Discovery of MBX-7591, a novel nonspirocyclic piperidine antifungal agent

To enable a cell-based screen using *A. fumigatus*, a mold that grows as a biofilm in assay plates, we utilized a strain that constitutively expresses firefly luciferase (24). Use of a luciferase-expressing *A. fumigatus* strain enabled us to measure the viability of cultures that have been exposed to screening compounds using a simple bioluminescence assay that is amenable to high-throughput screening (HTS). To identify potential inhibitors of the SrbA- pathway involved in triazole drug resistance, we included 32 µg/mL of fluconazole (FLC) with 10 µM of individual small molecules in the HTS. *A. fumigatus* is intrinsically resistant to fluconazole due to the presence of both *cyp51A* and *cyp51B* (25, 26). However, loss of *srbA* results in significant FLC susceptibility (15). Therefore, small molecules that target the *srbA* pathway are expected to increase susceptibility to fluconazole.

After screening over 206,000 compounds, we identified 29 verified hits that met the criteria of all secondary screens (Table S1). To prioritize hits, we evaluated the antifungal activity of each compound in a standard MIC assay. The structures and data for 16 selected hits are shown in Table S2. The prioritized hits comprise several core structures; however, the most potent hits have either the oxa-spirocyclic piperidine scaffold (compounds 2 and 3) or the nonspirocyclic piperidine scaffold (compounds 1, 4, 9, and 12). As expected, the MICs of the prioritized hits were significantly lower in the presence of 32 µg FLC/mL (Table S2), indicating the hit compounds potentiate the antifungal activity of FLC. Based on the antifungal activity and data from a panel of secondary assays designed to evaluate potency, selectivity, and drug-like activity, we selected compound 12 (MBX-7591) for further evaluation (Table 1).

**TABLE 1:** Structure of MBX-7591 and a summary of *in vitro* activities^*a*^

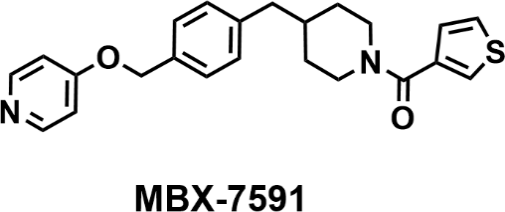	
Assay	Value
MBX-7591 MIC vs *A. fumigatus* (−) FLC (µM)	1.2
MBX-7591 MIC vs *A. fumigatus* (+) FLC (µM)	0.4
Ratio	3
Cytotoxicity (CC_50_ in μM)	≥100
MLMS (*t*_1/2_ in min)	40
Human CYP3A4 inhibition (%INH at 10 µM)	89
Caco-2 permeability (Papp, A→B/B→A)	1.1 × 10^−5^/5.6 × 10^–6^
Caco-2 efflux ratio	0.49
Aqueous solubility (μM)	50

^*a*^ CC_50_, half-maximal cytotoxic concentration; MLMS, murine liver microsome stability; Papp, apparent permeability coefficient.

### MBX-7591 spectrum of activity includes drug-resistant fungal species

To determine the spectrum of MBX-7591 antifungal activity, we measured MBX-7591 activity against other fungal species using CLSI-based microbroth dilution assays. MBX-7591 is active against a broad spectrum of filamentous fungal species (Table S3). Intriguingly, strain-specific activity was observed for some species, including *Apophysomyces* spp*., Saksenaea* spp.*, Blastomyces dermatiditis, Coccidioides* spp*.,* and *Histoplasma capsulatum*. Potent activity was observed against *Rhizopus delemar* strain 99–880 (~0.25 µg/mL) and various *A. fumigatus* strains (~2 µg/mL). Surprisingly, no activity was observed against other tested *Aspergillus* species (Table S3). Among the yeasts that were evaluated, only *Cryptococcus neoformans* was susceptible to MBX-7591. However, *C. neoformans* susceptibility was only observed when the organism was grown in the presence of 5% CO_2_ (Tables S3 and S4). *A. fumigatus* susceptibility to MBX-7591 was not impacted by CO_2_ levels.

### MBX-7591 potentiates triazole antifungal activity

**Fig 1:**
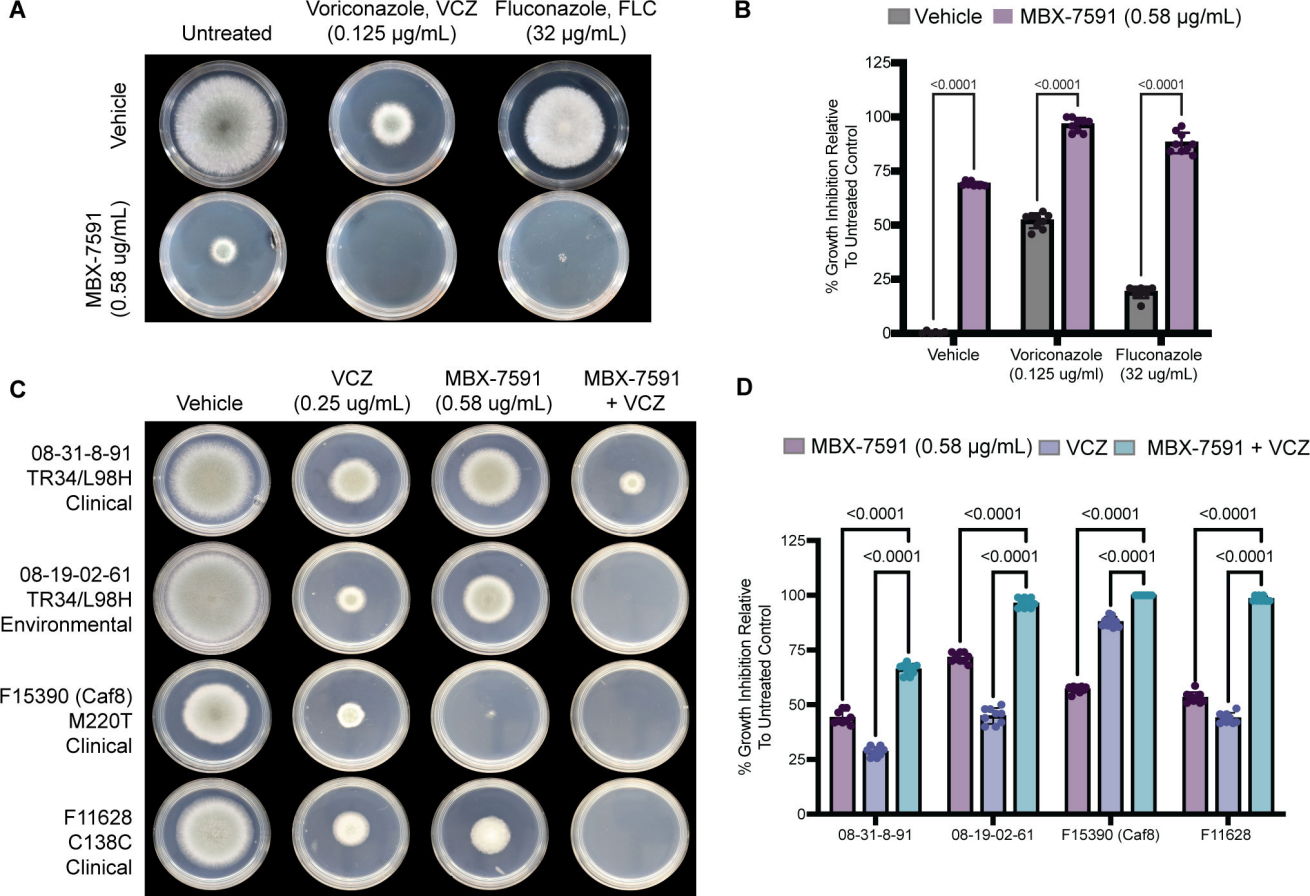
MBX-7591 has synergy with triazoles against laboratory, clinical, and environmental isolates in both conidial and biofilm-based assays. (**A and B**) MBX-7591 potentiates FLC and VCZ in agar-based colony biofilm assays. Plates were inoculated with 1 × 10^3^ conidia and incubated at 37°C, 5% CO_2_ for 72 hours. Representative images from three independent biological replicates with three technical replicates are shown. Colony diameter of each plate was collected and normalized to percent growth inhibition of the untreated control. Statistical significance between groups was determined through two-way ANOVA with Šídák’s multiple comparisons test. (**C and D**) MBX-7591 can sensitize triazole-resistant clinical and environmental isolates with different *cyp51A* mutations to VCZ in agar-based colony biofilm assays. Assay was performed and data were analyzed as described in panels B and C for three independent biological replicates with three technical replicates each.

To further define the ability of MBX-7591 to potentiate triazole (FLC and VCZ) activity against *A. fumigatus,* we utilized microbroth dilution checkerboard assays to assess the interaction between these triazoles and MBX-7591 (Table S5). A sub-MIC concentration (0.613 µg/mL) of MBX-7591 lowers the FLC MIC from >256 to 16 µg/mL, a >16-fold increase in FLC potency (FIC = 0.312) (Table S5). As *A. fumigatus* infections are primarily treated with VCZ, we tested if MBX-7591 has synergy with VCZ. Though VCZ is highly efficacious against most wild-type *A. fumigatus* strains (MIC: 0.25–0.5 μg/mL), the MIC of VCZ in combination with 0.613 µg/mL MBX-7591 is four to eight times more potent (MIC: 0.062–0.125 μg/mL), with an average FIC of 0.5 (Table S6). MBX-7591 potentiation of triazoles is also readily observed in agar-based colony biofilm assays and submerged biofilm assays (Fig. 1A and B; Fig. S1).

### MBX-7591 increases triazole susceptibility to triazole-resistant isolates

We next tested whether MBX-7591 potentiates triazole activity in *A. fumigatus* triazole-resistant strains using a panel of 12 distinct strains with diverse geographic origins and *cyp51A* alleles that confer pan-triazole resistance. We observed that none of these strains exhibited significant resistance to MBX-7591, as defined by greater than fourfold minimum inhibitory concentration compared to WT laboratory strains (Table S6). This result indicates a lack of genetic variants in these strains that confer resistance to MBX-7591 and that *cyp51A* allelic variants are not a determining factor for MBX-7591 susceptibility. To test if MBX-7591 increases triazole susceptibility in triazole-resistant strains, we selected three clinical strains with different *cyp51A/*Cyp51A genotypes (M220T, G138C, and TR34/L98H) and one environmental strain with the TR34/L98H genotype (27, 28). Using a checkerboard MIC assay, we observed that MBX-7591 potentiated VCZ against strains with the TR34/L98H genotype (08-31-08-91 and 08-19-02-61), in which MBX-7591 reduced the MIC of VCZ from 4 and 2 µg/mL, respectively, to 1 and 0.5 µg/mL (Table 2). The strain with an M220T *cyp51A* genotype (F15390) showed modest resistance to VCZ (MIC: 1 µg/mL), which was reduced fourfold to 0.25 µg/mL with the addition of MBX-7591 (Table 2). For strain F11628 (G138C *cyp51A* genotype), the VCZ MIC exceeded the concentrations we tested (>8 µg/mL) as previously reported (27). Remarkably, the addition of MBX-7591 decreased the MIC of VCZ by at least eightfold to 0.5–1 µg/mL for an FIC value of 0.3125–0.375 (Table 2). These results were further validated in agar-based colony biofilm assays as measured by the changes in colony diameter (Fig. 1C and D). Taken together, these results indicate that MBX-7591 is a small molecule with intrinsic antifungal activity that also potentiates the activity of the triazole class of drugs, regardless of strain *cyp51A* genotype and associated pan-triazole resistance.

**TABLE 2:** Summary of synergy assessment between voriconazole and MBX-7591 against azole-resistant isolates

Strain	MIC VCZ (µg/mL)	MIC MBX-7591 (µg/mL)	MIC VCZ in combination (μg/mL)	MIC MBX-7591 in combination (μg/mL)	FIC value
CEA10	0.25–0.5	2.453	0.0625–0.125	0.613	0.375–0.5
08-31-08-91	4	4.906	1	1.227	0.5
08-19-02-61	2	2.453	0.5	0.613	0.5
F15390 (caf8)	1	2.453	0.25	0.613	0.5
F11628	>8	2.453	0.5–1	0.613	0.3125–0.375

### MBX-7591 induces an increase in SrbA levels

**Fig 2:**
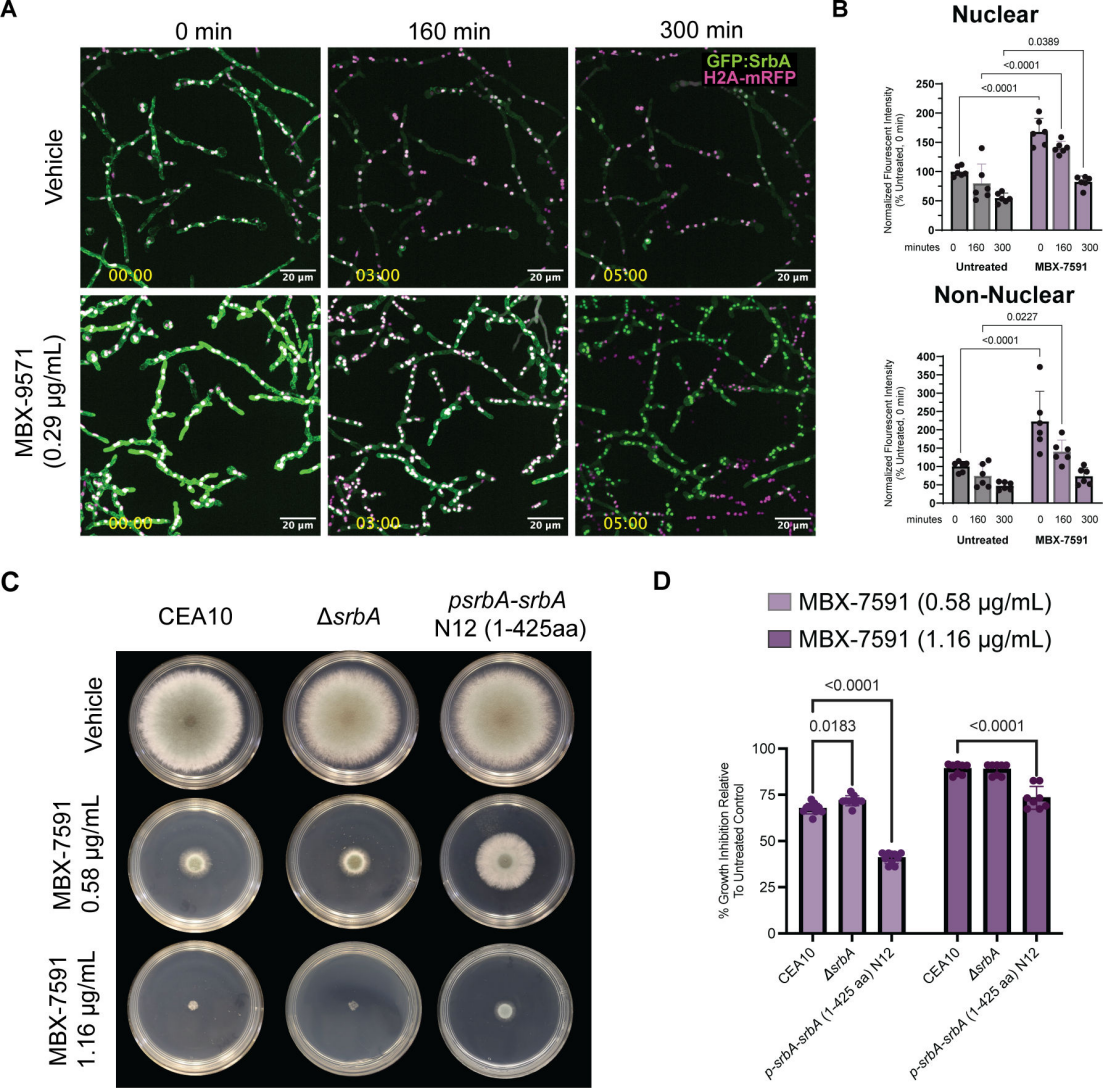
The transcription factor SrbA mediates susceptibility to MBX-7591. (**A**) Representative images of 5-hour microscopy timelapse of GFP-N-terminus-tagged SrbA 12-hour biofilms grown in vehicle or 0.29 µg/mL MBX-7591. Images were taken at minutes 0, 120, and 300. (**B**) MBX-7591 increases fluorescence intensity of GFP:SrbA in both nuclei (white) and non-nuclei (green) compared to vehicle control. Nuclear and non-nuclear GFP location was determined using the H2A-mRFP channel to select appropriate locations for analysis. Twenty locations per image per location (nuclear, non-nuclear, background), per image (three each), and per time point (0, 160, and 300 min) were measured for GFP intensity per biological replicate. The GFP intensity of each image at each location was averaged. Each image’s average was then normalized to the percentage of mean GFP intensity of the vehicle control at time point 0. These data represent two biological replicates, with three technical replicates each for a total of 120 locations measured per time point per condition. Statistical significance between groups was determined through two-way ANOVA with Šídák’s multiple comparisons test. (**C**) Activation of SrbA (*psrbA:srbA^1-425aa^*) decreases susceptibility to MBX-7591 in agar-based colony biofilm assays. Plates were inoculated with 1 × 10^3^ conidia and incubated at 37°C, 5% CO_2_ for 72 hours. Representative images from three independent biological replicates with three technical replicates are shown. (**D**) Colony diameter of each plate was collected and normalized to percent growth inhibition of their own untreated control. Statistical significance between groups was determined through two-way ANOVA with Šídák’s multiple comparisons test.

We next sought to determine whether the MBX-7591 mechanism of action involved the SrbA genetic network (17). Similar to MBX-7591 treatment, loss of *srbA* results in increased susceptibility to both FLC and VCZ in both wild-type and triazole-resistant strains (15, 20). Consequently, we next tested the hypothesis that MBX-7591 inhibits the activation of SrbA using an *A. fumigatus* strain expressing an SrbA N-terminal GFP-tagged allele and an H2A-mRFP nuclear marker to assess SrbA localization in the cell. Placing GFP on the N-terminus of SrbA allows tracking of both the full-length protein localized in the endoplasmic reticulum (ER) and the proteolytic processed N-terminus containing the basic helix loop helix (bHLH) transcription factor domain that localizes to the nuclei. In steady-state no-molecule/drug conditions, SrbA is observed as expected in both the ER and nuclei (Fig. 2A). We next grew 12-hour biofilms with vehicle control or a low-concentration of MBX-7591 (0.29 µg/mL, eightfold less than the MIC). After 12 hours of growth, we imaged biofilms every 20 min for a total of 5 hours (300 min) and analyzed the images to measure ER and nuclear GFP fluorescence. After growing the biofilms for 12 hours (time point 0), there is a clear difference in hyphal morphology in biofilms treated with a sub-MIC level of MBX-7591 (Fig. S2). Hyphae showed altered polarity, as evidenced by an increase in crooked and hyper-branching hyphae compared to the straight, narrow hyphae observed in the untreated group (Fig. S2). Additionally, there was a significant increase in both ER and nuclear GFP-SrbA signal in the MBX-7591 pre-treated sample (Fig. 2B). Nuclear GFP-SrbA signal was confirmed (white puncta) as co-localization with the H2A-mRFP histone marker. These data suggest that MBX-7591 increases the overall levels of SrbA in the cell and that proteolytic processing or localization of SrbA was not inhibited by sub-MIC levels of MBX-7591.

### SrbA levels influence MBX-7591 activity

The increase in SrbA cellular levels in response to MBX-7591 suggests that SrbA is involved in the cellular response to MBX-7591. To test this hypothesis, we asked whether the loss of *srbA* impacts MBX-7591 susceptibility using colony biofilm assays (Fig. 2C and D). The growth of the *ΔsrbA* strain in the presence of MBX-7591 (72%–89% of untreated) was not significantly different than that of the parental wild-type strain (~67% and ~89% of untreated), suggesting that sub-MIC levels of MBX-7591 do not directly inhibit SrbA.

We next asked whether a strain expressing the proteolytically processed N-terminus of SrbA containing the bHLH domain (N-SrbA, p*srbA:srbA*^1-425^), which constitutively localizes to the nucleus to regulate SrbA target gene transcription (29), exhibits altered susceptibility to MBX-7591. In contrast to the *srbA* null mutant strain, the N-SrbA strain had a 30% reduction in growth inhibition in the presence of 0.58 µg/mL MBX-7591 as compared to WT (Fig. 2C and D). At a higher concentration of MBX-7591, N-SrbA was able to maintain more growth compared to the wild-type strain (Fig. 2C and D). These data suggest that increased expression of SrbA results in decreased susceptibility to MBX-7591, possibly by increasing the intracellular concentration of the MBX-7591 molecular target. Taken together, these data support the hypothesis that MBX-7591 inhibits a target that is regulated by SrbA.

### AtrR mediates susceptibility to MBX-7591 through SrbA

**Fig 3:**
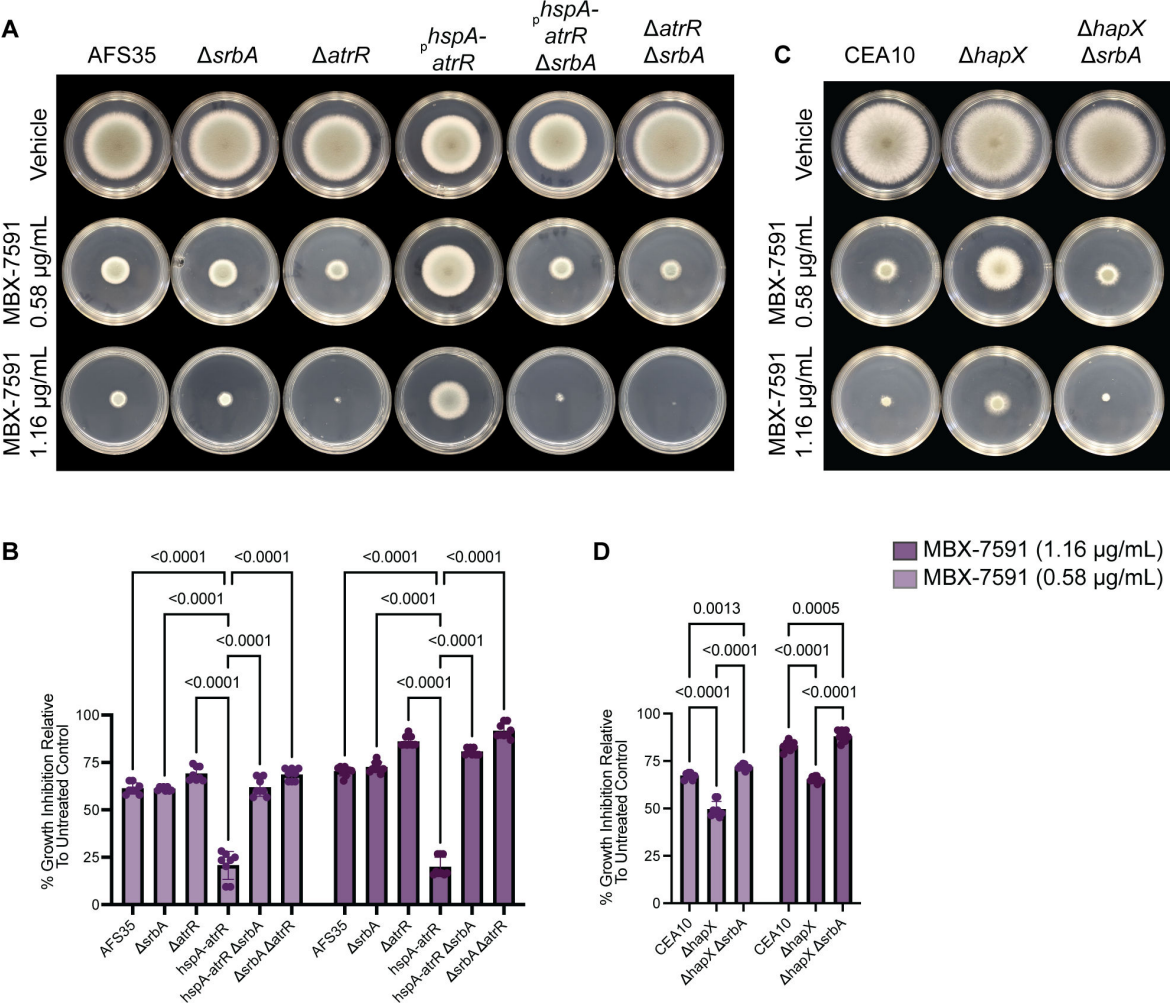
The transcription factors AtrR and HapX mediate susceptibility to MBX-7591 through SrbA. (**A**) Agar-based colony biofilm assay shows that *atrR* over-expression decreases susceptibility to MBX-7591 in a SrbA-dependent manner. Plates were inoculated with 1 × 10^3^ conidia and incubated at 37°C, 5% CO_2_ for 72 hours. Representative images from three independent biological replicates with three technical replicates are shown. (**B**) Colony diameter of each plate was collected and normalized to percent growth inhibition of their own untreated control. (**C**) Agar-based colony biofilm assay shows HapX loss decreases susceptibility to MBX-7591 in a SrbA-dependent manner. Plates were inoculated with 1 × 10^3^ conidia and incubated at 37°C, 5% CO_2_ for 72 hours. Representative images from three independent biological replicates with three technical replicates are shown. (**D**) Colony diameter of each plate was collected and normalized to percent growth inhibition of their own untreated control. Statistical significance between groups was determined through two-way ANOVA with Šídák’s multiple comparisons test.

One explanation for the modest change in MBX-7591 susceptibility in the absence of *srbA* is that the MBX-7591 target pathway is regulated by additional factors. There are two other transcription factors that co-regulate genes with SrbA: AtrR and HapX (18, 21, 22). AtrR is a transcription factor that also plays roles in low-oxygen adaptation, virulence, and triazole susceptibility (21, 22). AtrR co-regulates ~50% of SrbA target genes as measured by ChIP-seq (22). These genes include *srbA* itself and ergosterol synthesis pathway genes, such as *cyp51A* and *cyp51B*. Based on this overlap in gene regulation, we tested the role that AtrR plays in MBX-7591 susceptibility. We first confirmed that the parental strain, AFS35, for the *atrR* null mutant has similar susceptibility to MBX-7591 as CEA10 and AF293 (Table S6). Next, using our colony biofilm assay, we measured the susceptibility of the *ΔatrR* strain to MBX-7591 and observed a modest, concentration-dependent increase in MBX-7591 susceptibility as compared to *ΔsrbA* and AFS35 (Fig. 3A and B). Treatment of *ΔatrR* with MBX-7591 inhibits growth ~69% (0.58 μg/mL) and ~86% (1.16 μg/mL) compared to ~61% and ~70% in AFS35 and ~62% and ~72% in *ΔsrbA* at the lower and higher concentrations of MBX-7591, respectively. These data suggest that similar to SrbA, MBX-7591 is not directly inhibiting AtrR. However, in contrast to the *atrR* null mutant, a significant decrease in susceptibility to MBX-7591 is observed in a strain over-expressing AtrR with a strong promoter (p*hspA:atrR*) (Fig. 3A and B). MBX-7591 only inhibits growth ~9% (0.58 μg/mL) and ~19% (1.16 μg/mL) in the p*hspA:atrR* strain compared to the ~61% (0.58 μg/mL) and ~70% (1.16 μg/mL) observed in the parental strain AFS35. Taken together, these data suggest that similar to SrbA, AtrR expression levels influence MBX-7591 susceptibility.

In further support of SrbA-AtrR co-regulation of a pathway influencing MBX-7591 susceptibility, loss of *srbA* in the AtrR over-expression strain (p*hspA:atrR;ΔsrbA*) restored MBX-7591 susceptibility to almost wild-type levels (Fig. 3A and B). This result highlights that SrbA is necessary for AtrR-dependent MBX-7591 susceptibility. To test the hypothesis that SrbA and AtrR co-regulate the target or a gene responsible for mounting the cellular response to MBX-7591, we generated a *srbA* and *atrR* double null mutant strain. The *ΔsrbAΔatrR* strain showed a similar MBX-7591 susceptibility to the single Δ*atrR* strain at the lower dose of MBX-7591 (Fig. 3A and B). However, the increased growth inhibition at 1.16 µg/mL MBX-7591 of *ΔsrbA*Δ*atrR* (~92%) compared to Δ*atrR* (~86%) and *hspA-atrR;ΔsrbA* (~81%) supports a role for AtrR regulating the MBX-7591 target or cellular response to MBX-7591 directly (Fig. 3A and B). Overall, these data suggest that AtrR and SrbA mediate susceptibility to MBX-7591 through the regulation of the target and/or cellular response mechanism.

### HapX mediates susceptibility to MBX-7591 through negative regulation of SrbA

HapX negatively regulates *cyp51A* and *srbA* by directly binding their promoters (18, 30). As HapX negatively regulates *srbA* expression, we hypothesized that a Δ*hapX* strain would be less susceptible to MBX-7591. As expected, in the colony biofilm assay, loss of *hapX* resulted in a concentration-dependent decrease in MBX-7591 susceptibility compared to the parental CEA10 strain (Fig. 3C and D). To test whether the reduced MBX-7591 susceptibility in the Δ*hapX* strain was due to SrbA, we tested a *ΔsrbAΔhapX* double null mutant strain. As expected from our studies with SrbA, MBX-7591 susceptibility is restored to almost WT levels in *ΔhapX* when *srbA* is not present (Fig. 3C and D). Taken together, these data suggest the transcription factors SrbA, AtrR, and HapX regulate the MBX-7591 molecular target and/or induced cellular stress response.

### MBX-7591 has synergy with ergosterol synthesis pathway inhibition but does not target ergosterol synthesis genes

As HapX co-regulates gene expression with SrbA, we wondered how many genes co-regulated by AtrR and SrbA are also regulated by HapX (18). Thus, we compared genes identified as direct targets of HapX through ChIP-Seq analyses, in both iron-replete and deplete conditions, to the established AtrR/SrbA regulon of 51 genes also identified by ChIP-Seq analyses (22, 30). Comparing these genes across experiments identified 32 of the 51 genes in the AtrR/SrbA regulon as also regulated by HapX (Table S7). Gene Ontology (GO) analysis of these 32 genes utilizing the FungiDB platform revealed fatty acid biosynthetic processes as the most statistically significant (Bonferroni correct *P* value 0.0027) biological process with a 27.43-fold enrichment of these genes compared to the background genome (31, 32). Three of the four genes in the fatty acid biosynthesis GO term are involved in sterol biosynthesis and include genes encoding both C4 methylsterol oxidases Erg25A and Erg25B and the sterol delta 5,6 desaturase Erg3B. The fourth gene in this GO term encodes the stearoyl-CoA desaturase, SdeA. Four additional genes encoding enzymes involved in the ergosterol biosynthesis were also enriched among the 32 genes (Table S7).

These data suggested that MBX-7591 impacts ergosterol and/or fatty acid biosynthesis pathways and is consistent with the observed MBX-7591 potentiation of triazole antifungal activity. Therefore, we next tested inhibitors of multiple steps of the ergosterol synthesis pathway, including the allylamines, statins, and triazoles, and observed that MBX-7591 potentiates ergosterol pathway inhibition upstream of and at Cyp51 (Fig. S3A and B). Moreover, in an *erg5* null mutant and Δ*cyp51B;*pniiA:*cyp51A* strain that have reduced ergosterol levels, MBX-7591 susceptibility also increases (Fig. S3C and D). These data suggest reduced ergosterol levels in the cell potentiate MBX-7591 activity and likely rule out these ergosterol biosynthesis genes as direct targets.

### MBX-7591 changes cell membrane composition by decreasing oleic acid levels

**Fig 4:**
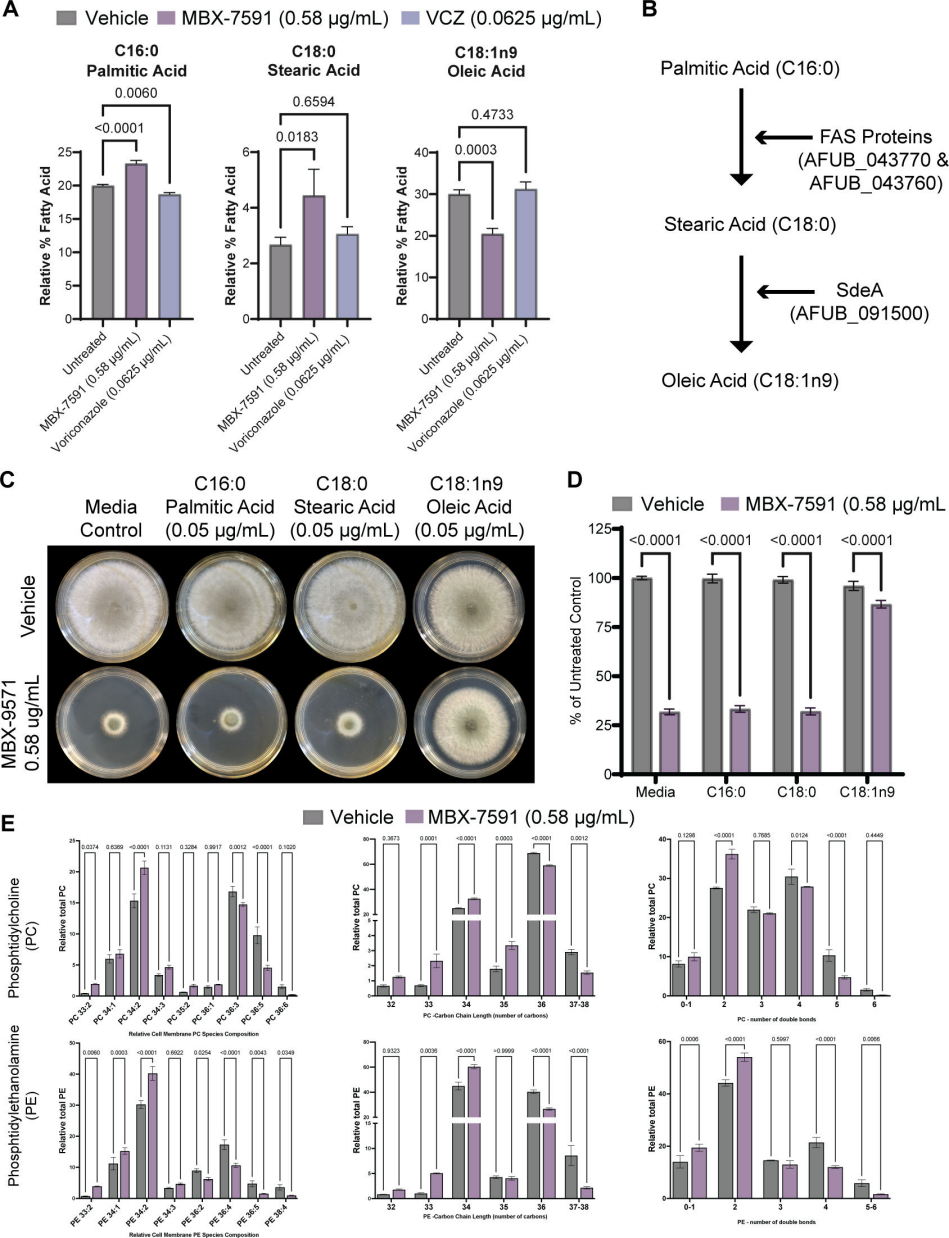
MBX-7591 perturbs cell membrane phospholipid composition through decreasing oleic acid content. (**A**) Relative percentage of palmitic, stearic, and oleic acid in 24-hour biofilms grown in the presence of vehicle control, 0.58 µg/mL MBX-7591, or 0.0625 µg/mL of voriconazole as assessed by GC-MS. MBX-7591 treatment increases the relative percentage of palmitic and stearic acid and reduces the relative percent of oleic acid compared to control. (**B**) Simplified model of the pathway from palmitic to oleic acid. (**C**) Addition of exogenous oleic acid greatly reduces susceptibility to MBX-7591, while palmitic acid and stearic acid do not in agar-based colony biofilm assays. Plates were inoculated with 1 × 10^3^ conidia and incubated at 37°C, 5% CO_2_ for 72 hours. Representative images from three independent biological replicates with three technical replicates are shown. (**D**) Colony diameter of each plate was collected and normalized to percent growth inhibition of their own untreated control. Statistical significance between groups was determined through two-way ANOVA with Šídák’s multiple comparisons test. (**E**) Analysis for intact phosphatidylcholine (PC) and phosphatidylethanolamine (PE) using HPLC-MS/MS. MBX-7591 treatment skews PC and PE populations to have shorter and more saturated fatty acid tails compared to controls.

As sterol biosynthesis inhibition appears to be an unlikely mechanism for the antifungal activity of MBX-7591, we turned to the observation that genes encoding proteins involved in membrane lipid composition, such as *sdeA,* that are part of the SrbA/AtrR/HapX genetic network could be a target of MBX-7591. To test this hypothesis, we measured changes in the relative abundance of fatty acids (FA) in 24-hour-old biofilm cultures grown in the presence of vehicle control, 0.58 µg/mL MBX-7591, or 0.0625 µg/mL of VCZ (Fig. 4A; Table 3). We observed that MBX-7591 treatment significantly altered the levels of three fatty acids: palmitic, stearic, and oleic acid (OA) (Fig. 4A; Table 3). These three FAs are metabolized in sequence to produce the monounsaturated FA oleic acid (Fig. 4B). Palmitic acid is converted to stearic acid through Fas1, then stearic acid is converted to OA through SdeA (also known as Ole1p in yeast). A sub-MIC MBX-7591 treatment thus increased saturated FAs palmitic and stearic acid and decreased the unsaturated FA oleic acid, suggesting oleic acid synthesis pathway inhibition (Fig. 4A).

**TABLE 3:** Summary of lipid profile results of CEA10 treated with MBX-7591 and voriconazole^*a*^

	C14:0	C15:0	C16:1n7	C16:0	C17a	C18:2n6	C18:1n9	C18:1n7	C18:0
Untreated	0.12 ± 0.017	0.15 ± 0.018	0.58 ± 0.022	**19.98** ± 0.19	0.18 ± 0.10	44.93 ± 0.99	**30.02** ± 1.03	1.36 ± 0.19	**2.68** ± 0.26
MBX-7591	0.19 ± 0.057	0.59 ± 0.034	0.68 ± 0.040	**23.30** ± 0.48	0.15 ± 0.025	48.87 ± 0.91	**20.48** ± 1.30	1.30 ± 0.18	**4.44** ± 0.95
Voriconazole	0.11 ± 0.0046	0.39 ± 0.11	0.38 ± 0.041	**18.71** ± 0.25	0.18 ± 0.15	45.91 ± 1.99	**31.26** ± 1.67	0.00 ± 0	**3.06** ± 0.26

^*a*^ In bold are palmitic, stearic, and oleic acid.

The stearoyl-CoA 9-desaturase, SdeA, is the single enzyme in *A. fumigatus* that modulates the first step of unsaturated FA synthesis and is a target of the SrbA/AtrR/HapX network (33). SdeA homologs in other fungal species are essential for viability (33–36). As the loss of *sdeA/OLE1* lethality is rescued with the addition of exogenous OA in other fungal species, we tested whether MBX-7591 susceptibility is rescued by exogenous OA (37). In our plate-based assay, the addition of 0.05 mg/mL of OA to the media almost fully restored the growth of CEA10 treated with a sub-MIC concentration (0.58 µg/mL) of MBX-7591 (Fig. 4C and D). We additionally confirmed that growth is rescued only by oleic acid and not by palmitic or stearic acid (Fig. 4C and D). We next utilized an FIC checkerboard assay with MBX-7591 and OA to assess their interaction. The addition of OA to the medium had a concentration-dependent antagonism to MBX-7591. The MIC for MBX-7591 without OA is 2.453 µg/mL, but with 15.625 µg/mL of OA, the MBX-7591 MIC increased eightfold to 19.625 µg/mL (Table S8). Further increasing OA concentration pushed the MBX-7591 MIC to higher levels than we measured. We also observed that OA antagonizes MBX-7591 in *Cryptococcus neoformans* at 5% CO_2_ (Table S8). This result supports MBX-7591 mediated unsaturated fatty acid synthesis pathway inhibition.

We next assessed if MBX-7591-mediated changes in lipid composition translated to changes in cell membrane phospholipid composition that would directly affect cell membrane fluidity. We repeated the experimental design from our lipid profiling assay but looked instead at the composition of the two most abundant cell membrane phospholipids, phosphatidylcholine (PC) and phosphatidylethanolamine (PE). Overall, MBX-7591 treatment increased the saturation and shortened the PC and PE fatty acid tail composition and length compared to untreated cells (Fig. 4E). This result agrees with the lipid profile as we saw a decrease in OA (C18n9) and an increase in the shorter and saturated FA palmitic acid (C16). As both phospholipid species were identically altered in their fatty acid tail composition, we conclude that these changes are due to MBX-7591-induced changes in the composition of available FAs.

Perhaps consistent with these lipid profiling data, transmission electron microscopy images of MBX-7591-treated cells reveal a loss of cellular membrane integrity, a large increase in the electron translucent cell wall, and a significant increase in electron density in the cytoplasm (Fig. S1B). These TEM images are consistent with cell death upon MBX-7591 treatment. To further test whether MBX-7591 is fungicidal, we examined colony formation after 48 hours of MBX-7591 treatment of conidia from CLSI MIC testing. No colony-forming units were observed at or 2× the MIC (Fig. S1C). Taken together, our data strongly suggest that MBX-7591 perturbs *A. fumigatus* cell membrane phospholipid composition leading to loss of cell viability. Moreover, these data suggest that the stearoyl-CoA 9-desaturase, SdeA, is a potential MBX-7591 molecular target.

### MBX-7591 can reach the site of infection and decrease fungal burden in mouse models of IPA

**Fig 5:**
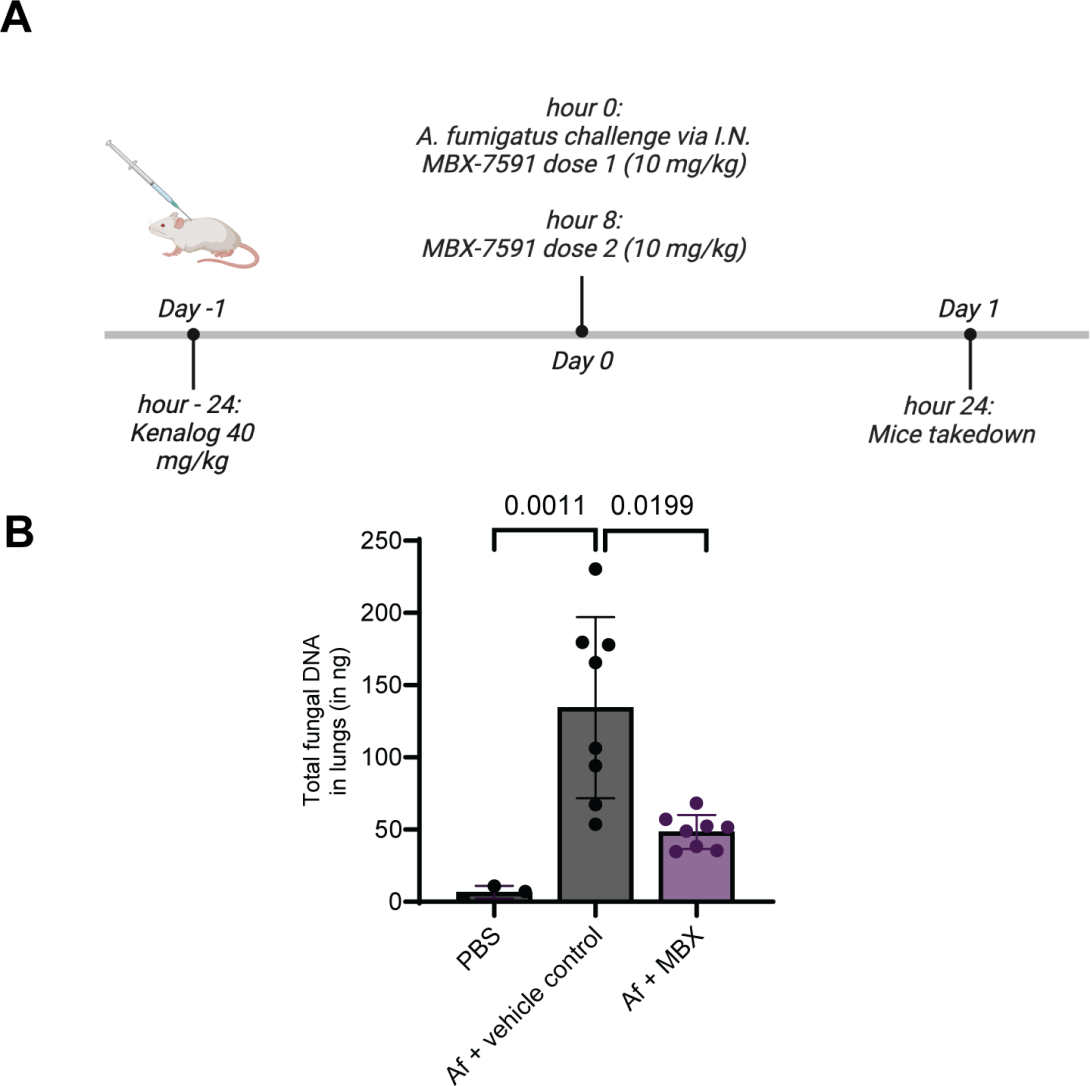
MBX-7591 is effective in decreasing fungal burden in murine models of invasive aspergillosis. (**A**) Experimental design of animal experiment. Mice were immunosuppressed using 40 mg Kenalog/kg of body weight (triamcinolone) 24 hours prior to intranasal inoculation with 2 × 10^6^ live conidia of *A. fumigatus strain* ATCC46445 or PBS control. After fungal inoculation, mice received an intraperitoneal injection of either vehicle control or 10 mg MBX-7591/kg of body weight. Second dose of either vehicle or MBX-7591 was administered 8 hours post-inoculation. Mice were humanely euthanized 24 hours post-infection, and lungs were collected for fungal burden assessment and histology. (**B**) *A. fumigatus* relative burden from mouse lungs was quantified with 18S rDNA qPCR assay 24 hours post-inoculation. Statistical significance was determined using ANOVA with the Kruskal-Wallis multiple comparison test.

Given the promising *in vitro* activity of MBX-7591, we next investigated some of its absorption, distribution, metabolism, and excretion (ADME) properties with the aim of conducting a preliminary animal model infection experiment. MBX-7591 does not exhibit cytotoxicity against mammalian cells [half-maximal cytotoxic concentration (CC_50_) >100 µM] and is stable in the presence of murine liver microsomes (*t*_1/2_ 40 min), which is a predictor of the half-life of the compound in mice (Table 1). While MBX-7591 exhibits inhibitory activity against the cytochrome P450 enzyme CYP3A4, this off-target activity is unlikely to interfere with a murine infection model. Given its expected low mammalian toxicity and reasonable stability in liver microsomes, we tested whether MBX-7591 could inhibit *A. fumigatus* growth *in vivo* in a murine model of invasive pulmonary aspergillosis. Mice were immunosuppressed with 40 mg triamcinolone/kg of body weight and were challenged via the intranasal route with *A. fumigatus* strain ATCC46645. A few minutes after fungal challenge, mice received 10 mg MBX-7591/kg of body weight via an intraperitoneal route. A second dose, 10 mg MBX-7591/kg of body weight, was delivered 8 hours later (Fig. 5A). Twenty-four hours after fungal challenge, lungs were harvested, and *A. fumigatus* relative burden was quantified with 18S rDNA PCR assay as previously described (38–40). Mice treated with MBX-7591 had an over threefold decrease in fungal burden compared to mice challenged with *A. fumigatus* but treated with the vehicle control (Fig. 5B). These data indicate that a relatively low dose of MBX-7591 likely reaches the murine lung and inhibits *A. fumigatus* growth.

## DISCUSSION

**Fig 6:**
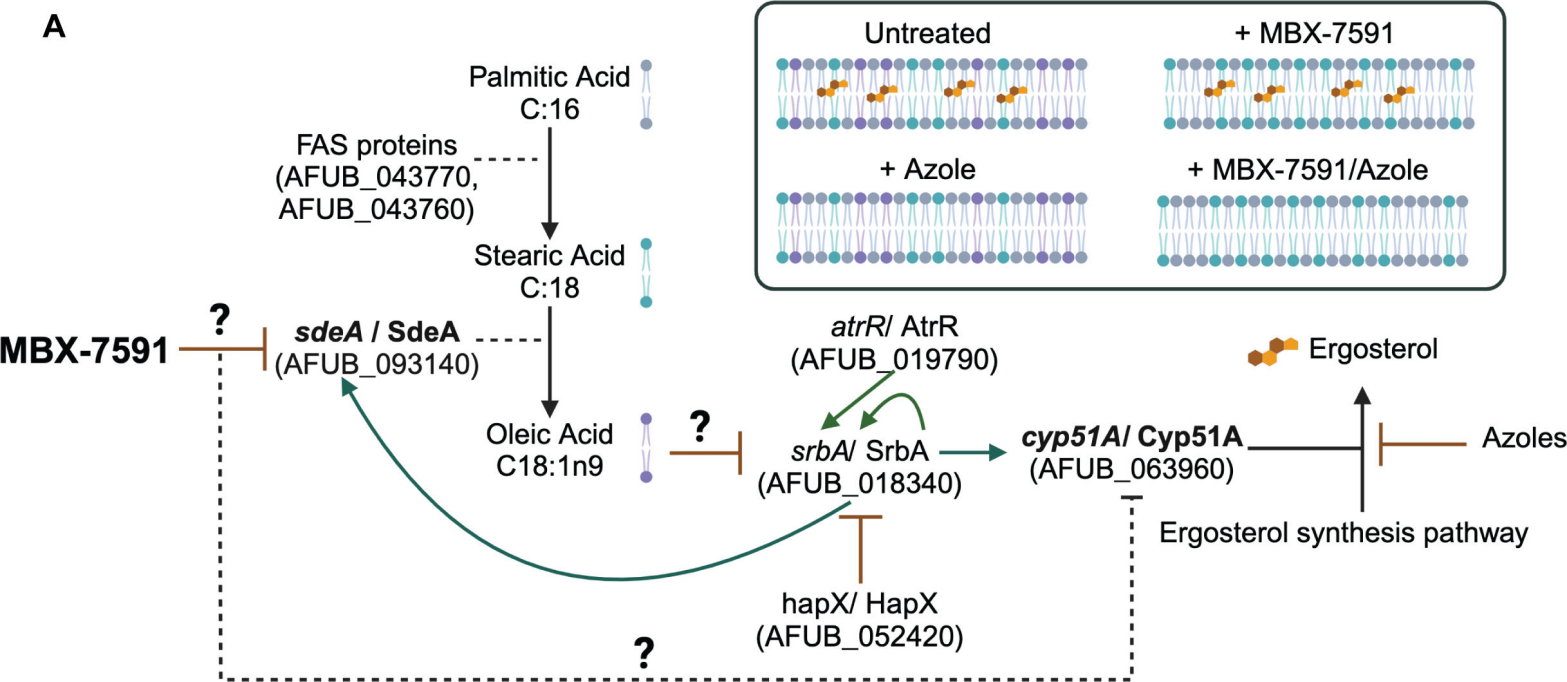
Proposed model of MBX-7591 antifungal activity. (**A**) Model of MBX-7591 mode of action. MBX-7591 directly or indirectly inhibits SdeA function, causing a decrease in oleic acid and an accumulation of precursors, palmitic and stearic acid. This shift in available FA alters cell membrane composition, causing phospholipids to have shorter and more saturated fatty acid tails. These changes in saturation in the cell membrane are potentially a signal sensed by transcription factor SrbA through an unknown mechanism to induce the activation of SrbA that results in an increase in *sdeA* transcription through SrbA. MBX-7591 susceptibility through SrbA activity can be modulated through transcription factors AtrR and HapX that regulate *srbA* in a positive and negative manner, respectively. Co-treatment of MBX-7591 and voriconazole results in increased cell membrane saturation. There is a possibility that inhibition of SdeA through MBX-7591 results in decreased levels of Cyp51A.

There is a dire need for novel antifungals to expand treatment options and combat increasing triazole resistance, particularly against filamentous fungi (5, 41). In this study, we identified a novel small molecule MBX-7591 that is effective at inhibiting the growth of *A. fumigatus* and other clinically relevant pathogenic fungi *in vitro* and *in vivo*. MBX-7591 has synergy with triazoles and increases voriconazole susceptibility in pan-azole-resistant isolates. While further mechanistic studies are needed to identify MBX-7591 molecular target(s), our data suggest that MBX-7591 increases the saturated:unsaturated fatty acid ratio in the fungal cell. One potential MBX-7591 molecular target candidate is the stearoyl-CoA 9-desaturase, SdeA (proposed model in Fig. 6). Previous and recent studies on SdeA function in fungi provide further support for the hypothesis that SdeA is a potential molecular target of MBX-7591. SdeA function was recently explored in *A. fumigatus* and found to be a conditionally essential gene (33, 42). Wang et al. (33) observed that loss of *sdeA* could partially be complemented with oleic acid and Tween 80, while Fabri et al. (42) generated a conditional *sdeA* expression strain that could not grow in the absence of the inducer (xylose) without the addition of oleic acid. We confirmed these findings with our own xylose-inducible *sdeA* strain (Fig. S4). Strikingly, our lipid profile of MBX-7591-treated *A. fumigatus* cells is highly similar to the lipid profile of the conditional *sdeA* strain characterized by Fabri et al. (42) highlighted by the increase in saturated phospholipids and decrease in monounsaturated fatty acids. Similar to oleic acid complementation of the *sdeA* genetic mutant strains, MBX-7591 antifungal activity is significantly reduced in the presence of oleic acid. Interestingly, Fabri et al. (42) reported that the *sdeA* conditional expression strain had wild-type levels of voriconazole and fluconazole susceptibility, which is in contrast to MBX-7591-treated *A. fumigatus* cells reported here (42). However, it is unclear under what assay conditions the Fabri et al. experiments were conducted. It is possible that the expression levels of *sdeA* were high enough in their assay to circumvent any enhanced triazole susceptibility, but it is also possible that MBX-7591 triazole synergy is not related to the inhibition of SdeA function.

The potent synergy observed between MBX-7591 and triazoles (and other ergosterol biosynthesis pathway inhibitors) is a defining feature of MBX-7591 and further supports a mechanism of action targeting membrane lipid composition. Altering the fatty acid composition of fungal cells has been previously observed to impact drug susceptibility in fungi. Intriguingly, studies of a *Saccharomyces cerevisiae* temperature-sensitive *ole1* mutant (the ortholog of *sdeA*) showed increased cell membrane saturation, which in turn induced Erg1p degradation as a way to reduce lanosterol accumulation (37). The authors hypothesized that lanosterol accumulation was due to reduced or impaired Erg11p (Cyp51 in *A. fumigatus*) function. Additionally, they observed that the *ole1* temperature-sensitive mutant had increased susceptibility to fluconazole at a semi-permissive temperature, supporting their hypothesis that decreased Ole1p (SdeA) activity results in decreased Erg11p (Cyp51 enzyme) activity (37). These tie-ins between Ole1p (SdeA) and Erg11p (Cyp51 enzymes) in *S. cerevisiae* provide one explanation for the synergy we observe with MBX-7591 and ergosterol biosynthesis pathway inhibitors that act upstream of Cyp51 enzymes (allylamine and statins). Regardless of the mechanism that remains to be defined, the potent synergy between MBX-7591 and triazoles is an exciting discovery that warrants further investigation in this era of emerging triazole resistance in *A. fumigatus*.

An interesting result from our MBX-7591 spectrum of activity screen is the lack of activity against other tested *Aspergillus* species. It seems clear that *A. fumigatus* possesses one functional copy of SdeA, as detailed by Fabri et al. (42) and confirmed here, which is in contrast with other *Aspergillus* species (Table S9). Thus, one possibility to explain the MBX-7591 resistance in other *Aspergillus* species, if SdeA is the molecular target, is an increase in functional *sdeA* copy number. For example, in *Aspergillus nidulans,* individual null mutants of *ΔsdeA* and *ΔsdeB* are viable, while the double null mutant is lethal (43). *A. nidulans ΔsdeA* and *ΔsdeB* lipid profiles had an increase in palmitic and stearic acid, with a significant decrease in oleic acid. These data are again similar to MBX-7591 treatment of *A. fumigatus*. We did not observe a significant reduction in susceptibility to MBX-7591 in our xylose-inducible *sdeA* strain, but we were unable to achieve expression levels higher than approximately threefold increase over wild-type levels despite repeated attempts (Fig. S4B and C). In addition to the differences in the copy number of stearoyl-CoA 9-desaturases across *Aspergillus* and other fungal species, the amino acid sequence similarity between species is highly variable. Thus, differences in MBX-7591 affinity for its target could also explain the divergent species susceptibility profiles we observed. Additional research is needed to definitively identify the molecular target of MBX-7591.

Our data also suggest that fungal species-specific responses to environmental conditions under which antifungal susceptibility testing is conducted may also contribute to our spectrum of activity data. An intriguing observation from our study is the CO_2_ concentration dependency of MBX-7591’s activity against the yeast *C. neoformans*. Recent studies in this important pathogenic yeast have highlighted the critical role of CO_2_ in pathogenesis and antifungal drug susceptibility (44–46). Under CO_2_ concentrations found in host tissue, *C. neoformans* susceptibility to triazoles is markedly increased (44). Here, under CLSI MIC microbroth dilution testing conditions that do not contain host-relevant concentrations of CO_2_, *C. neoformans* displays little susceptibility to MBX-7591. However, under CO_2_ concentrations found in host tissue, MBX-7591 has an MIC against *C. neoformans* similar to *A. fumigatus*. Intriguingly, CO_2_ environmental levels clearly alter membrane structure in *C. neoformans* (46). This is in line with our working hypothesis that MBX-7591 targets membrane lipid composition. It will be interesting in future studies to see if pathways driving the *C. neoformans* response to CO_2_ are involved in MBX-7591 drug susceptibility and how these are regulated and conserved across fungal species. Regardless, these data and observations further support the long-standing observation that environmental conditions have a significant impact on fungal membrane composition and drug susceptibility. Thus, we cannot rule out that the MBX-7591 spectrum of activity will increase with additional testing of less susceptible species under different environmental conditions more similar to those found *in vivo*.

Finally, an initial potential liability of MBX-7591 as a viable antifungal drug candidate is the inhibition of activity in the presence of oleic acid. It is unclear whether oleic concentrations *in vivo* in mice or human lungs are sufficient to inhibit MBX-7591 activity. However, Chayakulkeere et al. (47) observed that host fatty acid content is not able to rescue *C. neoformans* fatty acid synthesis defective mutants *in vivo*. It is promising that we observed efficacy *in vivo* in the IPA murine model at the initiation of the infection with no host-associated toxicity. If stearoyl-CoA 9-desaturase is the MBX-7591 target, the human ortholog has only 36.33% identity, and moreover, human stearoyl-CoA 9-desaturase has been suggested to be a promising anti-cancer target (48). Longer-term studies will be required to fully evaluate MBX-7591 *in vivo* and will also require detailed PK/PD before being feasible. To this end, MBX-7591’s chemical properties are amenable to lead optimization, which is ongoing in our laboratories with SAR studies. In conclusion, MBX-7591 is an exciting novel small molecule with antifungal properties and a potential novel mode of action against fungi historically associated with intrinsic and emerging resistance to contemporary antifungal drugs. Not only does MBX-7591 have lead development potential, but further study of its mechanism of action may reveal additional insights into new antifungal targets and/or potential avenues for improving existing drug efficacy against difficult-to-treat human fungal infections.

## MATERIALS AND METHODS

### Fluconazole potentiator screen

The HTS to identify MBX-7591 was conducted as described (24). Briefly, 25 µL of a spore suspension in optimized growth medium containing 32 µg fluconazole/mL was added to 384-well white assay plates (Thermo) containing 25 µL growth medium and screening compounds. The final concentration of screening compounds and DMSO was 10 µM and 1%, respectively. The plates were incubated at 35°C for 18–20 hours, after which bioluminescence reagent was added. After 30 min, the bioluminescence signal in each well was measured using an Envision plate reader (Perkin Elmer). Each plate contained a negative (0% inhibition) and a positive (100% inhibition) control comprising the inoculum treated with DMSO (1%) only or 32 µg FCL, respectively. The optimized parameters are as follows: growth medium, glucose minimal medium (GMM); incubation time, 18 hours; temperature, 37°C; spore density, 10^3^ spores/well; fluconazole concentration, 8× the MIC of the Δ*rbdB* strain (32 pg/mL); positive control, 4× the MIC of voriconazole (1 µg/mL) vs WT; and detection reagent, 5 µL of BrightGlo reagent (Promega) per well. Compounds that inhibited growth by ≥70% as compared to the negative control were scored as primary hits, which were confirmed in the screening assay with four technical replicates. The confirmed hits were evaluated for chemical tractability and the presence of known toxic substituents, and a subset of compounds was re-ordered from the vendor for further evaluation in secondary assays described below.

#### Antifungal activity assays

To assess antifungal activity, MICs were measured against the *A. fumigatus* CEA10 reference strain utilizing the standard CLSI microbroth dilution platform under the following conditions: (i) normoxia (20% O_2_, 5% CO_2_), and (ii) normoxia in the presence of 32 µg/mL fluconazole (49).

### ADME

#### Mammalian cell cytotoxicity (CC50)

The half-maximal cytotoxic concentration (CC50) of each compound against HeLa cells (ATCC CCL-2) was measured as previously described (50). Each assay comprised three technical replicates, and each assay was repeated at least once. The mean values for each biological replicate were averaged, and the CC50 was determined using a four-parameter nonlinear curve fitting algorithm (GraphPad Prism). The average of the CC50s from two biological replicates was calculated and reported.

#### Aqueous solubility

The maximum aqueous solubility of each compound was determined using a nephelometric method as described (51). Each assay comprised three technical replicates, and each assay was repeated at least once. The reported solubility is the average of at least five technical replicates.

#### Liver microsome stability

To examine the potential for first-pass metabolism of analogs in the liver, the stability of analogs in the presence of liver microsome preparations (Eurofins Discovery for human, dog, and rat; Xenotech for mouse) was measured using the method of Kuhnz and Gieschen (52). The amount of parent compound remaining after incubation with microsomes in the presence of NADPH over a 30-min time range was measured using a reverse-phase liquid chromatography/mass spectroscopy method that was customized for each compound. Half-lives were calculated using linear regression analysis of several time points.

#### CYP450 inhibition

Inhibition of the CYP450 enzyme CYP3A4 by each compound at a final concentration of 10 µM was measured using a commercially available Human Cytochrome P450 kit (BD Gentest Corp, Woburn, MA, USA) according to the manufacturer’s instructions.

#### Caco-2 permeability

To evaluate the potential for oral bioavailability, the ability of prioritized compounds to permeate a monolayer of Caco-2 intestinal epithelial cells was determined as described (53). Caco-2 permeability values (Papp) >1 × 10^–6^ cm/s are predictive of oral bioavailability. The observation that Papp A→B > Papp B→A indicates that efflux from the basolateral compartment does not occur.

#### Aqueous solubility

The maximum aqueous solubility of each compound was determined using a nephelometric method as described (51). Each assay comprised three technical replicates, and each assay was repeated at least once. The reported solubility is the average of at least five technical replicates.

### Antifungal susceptibility testing

#### Agar-based colony biofilm assays

All experiments were performed using glucose minimal media agar plates (6 g/liter NaNO_3_, 0.52 g/liter KCl, 0.52 g/liter MgSO_4_·7H_2_O, 1.52 g/liter KH_2_PO_4_ monobasic, 2.2 mg/liter ZnSO_4_·7H_2_O, 1.1 mg/liter H_3_BO_3_, 0.5 mg/liter MnCl_2_·4H_2_O, 0.5 mg/liter FeSO_4_·7H_2_O, 0.16 mg/liter CoCl_2_·5H_2_O, 0.16 mg/liter CuSO_4_·5H_2_O, 0.11 mg/liter (NH_4_)6Mo_7_O_24_·4H_2_O, 5 mg/liter Na_4_EDTA, 1% glucose; pH 6.5, 1.5% agar) supplemented with appropriate vehicle control or with any of these additives: MBX-7591 (0.58, 0.16 µg/mL), fluconazole (32 µg/mL), voriconazole (0.125, 0.5 µg/mL), fluvastatin (0.5 µg/mL), terbinafine (0.125 µg/ml), amphotericin B (0.5 µg/mL), palmitic acid (0.05 mg/mL), stearic acid (0.05 mg/mL), oleic acid (0.05 mg/mL), or a combination of MBX-7591 and any of the above. Plates were inoculated with 1 × 10^3^ spores in 2 µL and incubated for 72 hours at 37°C, 5% CO_2_. Colony diameter was measured using a ruler, and data were normalized to the appropriate untreated control and presented as percent growth. Images are representative of at least three independent biological replicates.

#### FIC checkerboard synergy and CLSI-based microbroth dilution assays for *Aspergillus fumigatus*

All antifungal susceptibility testing was done in 96-well plates using RPMI 1640 media buffered with 165 mM of MOPS and pH 7 using NaOH. CLSI-based microbroth dilution assays were done in accordance with M38 (49). Serial dilutions of the compound tested (voriconazole, MBX-7591) were done in the *Y*-axis of the plate, encompassing testing of 10 different concentrations. After serial dilutions, each well contained 100 µL of 2× of the desired final compound testing concentration. Each testing well was then inoculated with 100 µL of a stock of 5× 10^4^ spores/mL of the appropriate strain, for a final concentration of 2.5 × 10^4^ spores and to achieve the desired final compound testing concentrations. Additionally, an untreated control (100 µL of a stock of 5 × 10^4^ spores/mL and 100 µL of media) and a media-only control (200 µL media) were included in the plate. The plate was covered in a Breathe-Eazy membrane to allow for uniform oxygen diffusion throughout the plate and incubated for 48 hours at 37°C, 5% CO_2_. The minimal inhibitory concentration was determined by visually examining the plate and reported as the smallest compound concentration that completely inhibited growth.

For checkerboard synergy assays, serial twofold dilutions in the *Y*-axis were done with either voriconazole, fluconazole, or oleic acid for 4× the desired final concentration in 50 µL per well. Serial twofold dilutions of MBX-7591 were done in the *X*-axis for a 4× of the desired final concentration in 50 µL per well. Single-treatment controls were completed with 50 µL of media to substitute the volume of the other compound. Each well was then inoculated with 100 µL of a stock of 5 × 10^4^ spores/mL of the appropriate strain, for a final concentration of 2.5 × 10^4^ spores and to achieve the desired final compound testing concentrations. Additional untreated and media-only controls were added to the plate as described above. Incubation of the plates and MIC assessments (individual and in combination) were performed as described above. FIC calculations were done according to the established formula: $\sum FIC=\frac{MIC\;combination\;compound\;A}{MIC\;individual\;compound\;A}$ + $\frac{MIC\;combination\;compound\;B}{MIC\;individual\;compound\;B}$. Synergy was considered equal or lower than 0.5, and antagonism was considered to be higher than 2. FIC scores reported represent between three and five biological replicates.

#### CLSI-based microbroth dilution assays for yeast

All antifungal susceptibility testing was done in 96-well plates using RPMI 1640 media buffered with 165 mM of MOPS and pH 7 using NaOH. CLSI-based microbroth dilution assays were done in accordance with M27 (49). Serial dilutions of the compound tested (voriconazole, MBX-7591) were done in the *Y*-axis of the plate, encompassing testing of 10 different concentrations. After serial dilutions, each well contained 100 µL of 2× of the desired final compound testing concentration. Each testing well was then inoculated with 100 µL of a stock of 2 × 10^3^ spores/mL of the appropriate strain, for a final concentration of 1 × 10^3^ spores/mL and to achieve the desired final compound testing concentrations. Additionally, an untreated control (100 µL of a stock of 2 × 10^3^ spores/mL and 100 µL of media) and a media-only control (200 µL media) were included in the plate. The plate was covered in a Breathe-Eazy membrane to allow for uniform oxygen diffusion throughout the plate and incubated for 24 hours at 35°C (*Candida* spp.) or 72 hours at 35°C ± 5% CO_2_ (*Cryptococcus neoformans*). Minimal inhibitory concentration was determined by visually examining the plate and reported as the smallest compound concentration that completely inhibited growth.

### Fluorescent microscopy

#### Sample preparation

All microscopy experiments were conducted using liquid glucose minimal media (LGMM) autoclaved and filter sterilized supplemented with vehicle control or MBX-7591 (0.29 µg/mL). All microscopy experiments used a fluorescent strain of *A. fumigatus* with a GFP N-terminally tagged SrbA and an RFP histone tag (GFP::SrbA, H2A::mRFP) (29, 54). To generate the double-labeled strain, the H2A::mRFP construct that includes the *ptrA* pyrithiamine resistance gene was PCR amplified from the CEA10 H2A::mRFP strain with primers that contained homology to the *aft4* genomic safe haven site (54, 55). The fragment was transformed into the CEA10 GFP::*srbA* strain resulting in CEA10 GFP::*srbA*, H2A::mRFP.

#### GFP-SrbA level determination in MBX-7591-grown biofilms

To determine GFP-SrbA levels, 1 × 10^4^ spores/mL were grown on LGMM or LGMM containing 0.58 µg/mL MBX-7591 for a final concentration tested of 0.29 µg/mL in Ibidi µ-Slide 8 well glass bottom dishes (Ibidi GmbH, Germany). Cultures were incubated at 37°C, 5% CO_2_ for 12 hours to initiate biofilm growth. At the 12-hour time point, a timelapse was started, with images in the DIC, GFP, and RFP channels being taken every 20 min for a total of 5 hours (300 min). Imaging was performed on a spinning disk confocal microscope at 60× magnification using a *z*-depth of 21 µm (0.6 µm per z-slice). For each biological replicate, three technical replicate images per group were analyzed using Fiji imaging software (56, 57) For analysis, image intensity was measured from the sum of projected images at 20 locations per image at three time points, 0, 160, and 300 min. The intensity of the non-nuclear and nuclear GFP signal was measured using a 13-pixel diameter ROI. Non-nuclear and nuclear GFP localization was determined by monitoring the RFP channel. An adjacent background location was measured for each position to remove the background resulting in “normalized mean fluorescence intensity.” The mean for each image/time point was averaged resulting in “mean normalized mean fluorescence intensity.” To incorporate both biological replicates, the means of each image/time point/condition were normalized to the percentage of mean normalized mean fluorescence intensity of untreated at time point 0 min. These data represent two biological replicates for a total of 120 locations per condition per time point.

### Lipid and phospholipid profile

#### Fungal sample preparation for intact fatty acid and intact phospholipid analysis

Using WT laboratory strain CEA10, we inoculated 1 × 10^5^ spores/mL in 20 mL of LGMM in a sterile 100 mm petri plate. Petri plates were treated with either MBX-7591 (0.58 µg/mL), voriconazole (0.0625 µg/mL), or neither and incubated for 24 hours at 37°C, 5% CO_2_. Biomass was collected from three plates and pooled to form one technical replicate per condition, for a total of three technical replicates each. After collecting biomass in a 2 mL screw-cap tube, samples were immediately flash frozen in liquid nitrogen and stored at −80°C until ready to be processed.

#### Lipid extraction and analysis for intact fatty acid using GC/MS

Fungal samples grounded in liquid nitrogen were transferred to 1 mL methanol: sulfuric acid (975:25 µL) containing the internal standard 1,2-diundecanoyl-sn-glycero-3-phosphocholine (Avanti Polar Lipids) for total fatty acid extraction. The resulting solution was baked for 1 hour at 80°C to generate fatty acid methyl esters (FAMEs). A volume of 1.5 mL of water and 200 µL of hexane were then added to the samples and vortexed vigorously. FAMEs were recovered by snap freezing the aqueous layer and transferring the hexane layer into a vial for GCMS analysis.

For fatty acid analysis, 1 µL of FAMEs was injected into GC-MS (Thermo Trace 1310 GC with an ISQ LT single quadrupole mass spectrometer). The fatty acids were then separated by 5%-phenyl-methylpolysiloxane phase capillary column (HP-5ms, Agilent), and the eluted fatty acids were fragmented with an electron impact source for mass spectrometry analysis. The area under the peaks for each fatty acid was determined using the Thermo Fisher software Chromeleon (version 7.2.10 ES). The exported area was used to calculate the relative percentage of FAs in each sample (58).

#### Lipid extraction and analysis for intact phospholipids using HPLC-MS/MS

Fungal samples were ground in liquid nitrogen with a mortar and pestle. Total lipids were extracted from the tissue using 4 mL of chloroform:methanol (2:1) spiked with the internal standard 1,2-diundecanoyl-sn-glycero-3-phosphocholine (Avanti Polar Lipids) for 1.5 hours. Debris was then separated from the lipids by adding ~800 µL of 0.9% NaCl. The separated lipids were then dried down under a stream of nitrogen and resuspended in 200 µL of acetonitrile/2-propanol/water (65:30:5) for analysis.

For data acquisition on the Dionex UHPLC-MS/MS, 10 µL of the sample was injected, and the phospholipids were separated on a reverse phase column C18 Hypersil Gold (2.1 × 50 mm, 1.9 µm column) at a flow rate of 0.3 mL/min. A gradient elution with mobile phase A (60:40, water:acetonitrile, 10 mM ammonium formate, and 0.1% formic acid) and mobile phase B (90:10, isopropyl alcohol:acetonitrile, 10 mM ammonium formate, and 0.1% formic acid) was used with the following schedule: 32% B for 0–1.5 min, 45% B at 4 min; 52% B at 5 min; 58% B at 8 min; 66% B at 11 min; % B at 14 min; 75% B at 18 min; 97% B from 18 to 25 min, and finally 32% B from 25 to 30 min for column equilibration (59).

Phospholipids separated by chromatography were then analyzed by full MS data-dependent acquisition using a Quadrupole-Q Exactive mass spectrometer (Thermo Scientific, Waltham, MA, USA). The phospholipids were acquired in the negative ion mode at a scan range of *m/z* 300–1,200 and a resolution of 70, 000. The HESI capillary temperature was set at 325°C, the sheath gas flow rate was set at 45 units, the auxiliary gas flow was set at 10 units, the source voltage was 3.2 kV, and the AGC target was 106. MS2 analyses were performed using six scan events, where the top five ions were chosen from an initial MS1 scan for fragmentation at a normalized collision energy of 35. MS1 spectra were collected in profile mode, and MS2 spectra were collected in centroid mode.

Lipid data analyzer software (version 2.8.1) was used to quantify the area of each phospholipid based on the mass list. A 0.1% cutoff was used in order to focus on the major phospholipids (60).

### *In vivo* efficacy of MBX-7591 in murine model of invasive pulmonary aspergillosis

Outbred wild-type CD-1 female mice at 20–24 g (Charles River Laboratory, Raleigh, NC, USA) were randomized and housed in autoclaved cages at ≤4 mice per cage with HEPA-filtered air and water. Mice were immunosuppressed by subcutaneous injection with triamcinolone acetonide (Kenalong-10, Bristol-Myer Squibb) at 40 mg/kg of body weight. Twenty-four hours after immune suppression, mice were intranasally inoculated with 2 × 10^6^ live conidia (in 40 µL of sterile PBS) or PBS alone as a control. *A. fumigatus* strain ATCC46645 was grown on 1% GMM for 3 days at 37°C, conidia were collected in 0.01% Tween, and washed three times in sterile PBS. Immediately after fungal inoculation, mice were intraperitoneally (IP) injected with either vehicle (5% DMSO/10% PS80/25% HPBCDX 0.07% sodium acetate) or MBX-7591 at 10 mg/kg of body weight (5% DMSO/10% PS80/25% HPBCDX 0.07% sodium acetate). Mice were injected IP with a second dose of vehicle or MBX-7591 at 10 mg/kg of body weight 8 hours after the first dose of the drug. Mice were sacrificed 24 hours post-fungal challenge, lungs were excised from the chest cavity, rinsed in sterile PBS, and flash frozen for fungal burden analysis. Relative fungal burden was assessed through qPCR quantitation of *A. fumigatus* 18S rDNA as previously described (38–40).

## Data Availability

All underlying data are available either in the supplemental material or upon request to the corresponding author.

## SUPPLEMENTAL MATERIAL

Supplemental material mbio.01166-24-s0001.pdf Supplemental figures, tables, and text.

ASM does not own the copyrights to Supplemental Material that may be linked to, or accessed through, an article. The authors have granted ASM a non-exclusive, world-wide license to publish the Supplemental Material files. Please contact the corresponding author directly for reuse.
