# Supplementary material for: Unsaturated fatty acid perturbation combats emerging triazole antifungal resistance in the human fungal pathogen Aspergillus fumigatus
Source: mBio. 2024 Jun 27;15(7):e01166-24. doi: 10.1128/mbio.01166-24 (PMC11253624; doi:10.1128/mbio.01166-24)
Supplement: Supplemental material — Supplemental figures, tables, and text. [file mbio.01166-24-s0001.pdf]

Supplementary Information

SUPPLEMENTARY TABLES:

Supplementary table 1. Summary of the HTS for small molecules that inhibit intrinsic resistance to fluconazole in *Aspergillus fumigatus*.

**Screening stage**	**Number of compounds**	**% total**
Total screened	206240	100
Primary hits	1266	0.614
Med chem pass	1203	0.583
Confirmed hits	236	0.114
Reordered from vendor	53	0.026
MIC ≤ 10 μM (with 32 μg/ml FLUC)	39	0.019
**MIC-FLC/MIC+FLC ≥4**	**29**	**0.014**

Supplementary table 2. Summary of hits for small molecules that inhibit intrinsic resistance to fluconazole in *Aspergillus fumigatus*.

**Compound #**	**Structure**	**MIC (-Fluc) μM**	**MIC (+Flu) μM**	**Fold MIC**
**1**	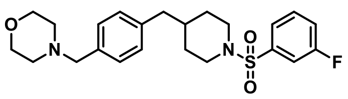	6.25	0.39	15.9

**2**	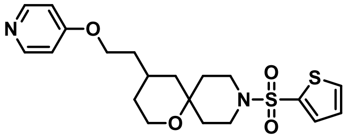	6.25	0.78	8
**3**	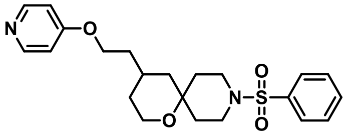	3.12	0.39	7.9
**4**	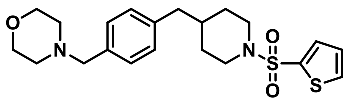	7.87	0.78	10
**5**	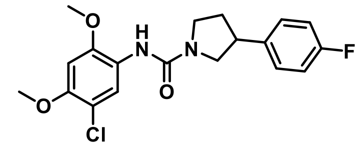	12.5	15.7	0.79
**6**	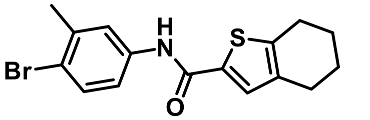	200	6.25	32
**7**	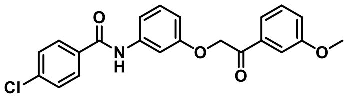	1.24	0.39	3.1
**8**	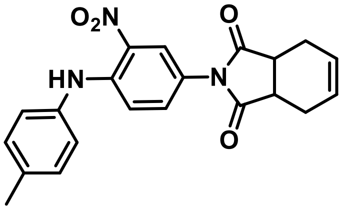	6.25	3.12	2
**9**	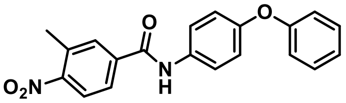	10	2.5	4

**10**	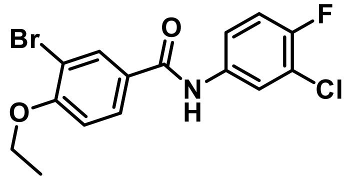	≥40	5	≥8
**11**	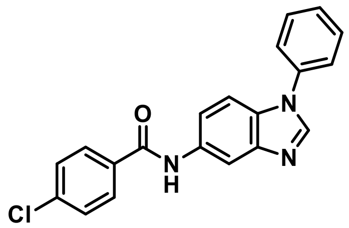	≥40	2.5	≥16
**12 (7591)**	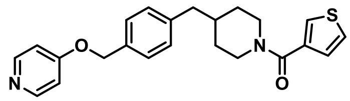	20	5	4
**13**	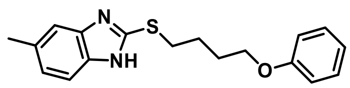	≥40	10	≥4
14	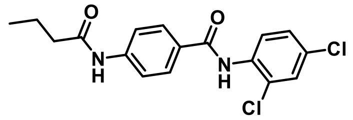	≥40	5	≥8
15	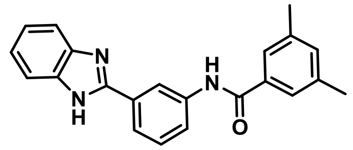	≥40	2.5	≥16
16	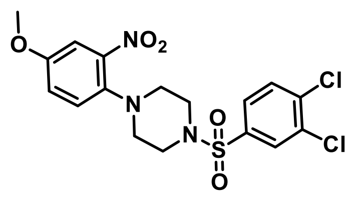	20	2.5	8

Supplementary Table 3. Selected results from MIC assessment of MBX-7591 against different fungal species between Cramer lab and University of Texas Health Science Center at San Antonio (preclinical services funded by the National Institute of Allergy and Infectious Diseases)

Species	Strain	MIC (µg/mL) Cramer Lab	MIC (µg/mL) UTHSC
Rhizopus delemar	***99-880***	**0.31**	**0.25**
Cryptococcus neoformans	*H99*	2.45*	>32
P. variotii	MYA-3630	N/A	>32
Apophysomyces spp.	***Apo1***	**N/A**	**0.5**
	Apo2		**16**
	Apo3		**4**
***Saksenaea* spp.**	**Sak1**	**N/A**	**0.5**
Blastomyces dematitidis	**DI19-271**	**N/A**	**0.5**
	**DI19-277**		**8**
	**DI19-282**		**4**
Coccidioides spp.	**DI14-269**	**N/A**	>32
	**DI14-284**		**8**
	**DI17-143**		>32
Histoplasma capsulatum	**DI14-269**	**N/A**	**4**
	**DI14-284**		**1**
	**DI17-143**		**0.5**
Aspergillus fumigatus	***CEA10, AFS35, AF293***	**2.45**	**N/A**
	AF293	**2.45**	**2**
	ATCC46455	**2.45**	**N/A**
Aspergillus flavus	*ATCC 204304*	N/A	>32
	Aflav1		>32

	Aflav2		>32
Aspergillus niger	*AN1*<br>*AN2*	N/A	>32<br>>32
Aspergillus terreus	*AT1*	N/A	>32
Aspergillus nidulans	*FGSC A4*	> 39.25	N/A
Candida albicans	*SC5314*	> 39.25	>32
Mucor spp.	*CBS 277.49*<br>*Mucor1*<br>*DI15-131*<br>*Mucor2*	> 39.25<br>N/A<br>N/A<br>N/A	N/A<br>>32<br>>32<br>>32
Fusarium spp.	*solani and oxysporum*	> 39.25	>32
Scedosporium spp.	*Cramer Lab: apiospermum (7D7), prolificans (7D8), JI 2018 "typical" (14E6)*<br>*NIH: DI19-67, DI15-15, DI19-66*	> 39.25	>32
Candida auris	*Cramer Lab:Clade I, Clade III*<br>*NIH: DI17-47, DI17-48, DI17-46*	> 39.25	>32

*MIC obtained under 5% CO_2

Supplementary table 4 – *Cryptococcus neoformans* susceptibility to MBX-7591 at different CO_2 concentrations

Strain ID	**MIC 0% CO_2 (µg/ml)**	**MIC 5% CO_2 (µg/ml)**
H99	> 39.25	2.45
A7-35-23	> 39.25	2.45
JEC21	> 39.25	2.45
C23	> 39.25	2.45

Supplementary table 5. Summary of FIC values of MBX-7591 and triazoles fluconazole (FLC) and Voriconazole (VCZ) in CEA10

CEA10	**MIC Azole (µg/mL)**	**MIC MBX-7591 (µg/mL)**	**MIC Azole in combination (µg/ml)**	**MIC MBX-7591 in combination (µg/ml)**	**FIC score**
FLC	>256	2.45	16	0.613	0.312
VCZ	0.25 – 0.5	2.45	0.0625 – 0.125	0.613	0.375 – 0.5

Supplementary table 6. Summary of triazole resistant isolate susceptibility to MBX-7591

Strain ID	Type of isolate	Geographic location	Triazole susceptibility	*cyp51A* genotype	Disease Type	Voriconazole MIC (µg/ml)	MIC MBX-7591 (µg/ml)
CEA10, AF293, AFS35 ATCC46455	Laboratory WT	N/A	N/A	N/A	N/A	0.25 – 0.5	2.45<br>1.23 – 2.45
RBH-AF-01	Clinical isolate	England	Resistant	Unknown	CF isolate	0.25	2.45
RBH-AF-02	Clinical isolate	England	Resistant	Unknown	CF isolate	2	2.45
RBH-AF-03	Clinical isolate	England	Resistant	Unknown	CF isolate	0.25	2.45
RBH-AF-08	Clinical isolate	England	Resistant	Unknown	CF isolate	2	2.45
RBH-AF-10	Clinical isolate	England	Resistant	Unknown	CF isolate	1	1.23
10-19-02-27	Clinical isolate	Netherlands	Resistant	TR34/L98H	COPD isolate	4	2.45
08-31-08-91	Clinical isolate	Netherlands	Resistant	TR34/L98H	Left Ear, Otis externa	4	4.91
08-19-02-10	Environmental Isolate	Netherlands	Resistant	TR34/L98H	N/A	2	4.91
08-19-02-46	Environmental Isolate	Netherlands	Resistant	TR34/L98H	N/A	2	2.45
08-19-02-61	Environmental Isolate	Netherlands	Resistant	TR34/L98H	N/A	1	2.45
F15390	Clinical isolate	Manchester, UK	Voriconazole resistant	M220T	CCPA with aspergilloma, then CFPA – clinical failure	1	2.45
F11628	Clinical isolate	Liverpool, UK	Voriconazole resistant	G138C	CCPA with bilateral aspergillomas, CFPA --clinical failure	>8	2.45

Supplementary Table 7. Summary of genes that are co-regulated by SrbA/HapX/AtrR according to ChIP-Seq

Gene ID	Function
AFUB_001370	Protein of unknown function
AFUB_004020	Orthologs have domain(s) with predicted heme binding, iron ion binding, oxygen binding activity and role in oxygen transport
AFUB_004130	Ortholog(s) have endoplasmic reticulum, mitochondrion localization, has domain(s) with predicted membrane localization
AFUB_004350	Cytochrome P450 sterol C-22 desaturase, putative (Erg5)
AFUB_009760	Putative phosphoglycerate kinase
AFUB_012310	Putative nitrite reductase
AFUB_018340	Sterol Regulatory Element Binding Protein Family member (SrbA)
AFUB_029460	Protein of unknown function. Has predicted domain of S-adenosyl-L-methionine-dependent methyltransferase
AFUB_034630	Has domain(s) with predicted DNA binding, zinc ion binding activity, role in transcription, DNA-templated and nucleus localization
AFUB_034690	Putative integral plasma membrane heat shock protein
AFUB_047590	Putative cholestenol delta-isomerase
AFUB_051820	CID domain-containing protein
AFUB_052310	Putative high-affinity iron permease. Has domain(s) with predicted role in transmembrane transport and membrane localization
AFUB_054020	Has domain(s) with predicted ATP binding, protein kinase activity, protein tyrosine kinase activity and role in protein phosphorylation
AFUB_055750	Protein of unknown function
AFUB_058270	Has domain(s) with predicted FMN binding, pyridoxamine-phosphate oxidase activity and role in oxidation-reduction process, pyridoxine biosynthetic process
AFUB_058280	Pentatricopeptide repeat protein
AFUB_062460	Putative phosphoenol pyruvate synthase
AFUB_063960	Sterol 14-alpha demethylase (Cyp51, Erg11)
AFUB_067210	Has domain(s) with predicted oxidoreductase activity and role in oxidation-reduction process
AFUB_068280	Putative carbonic anhydrase. Has domain(s) with predicted zinc ion binding, carbonate dehydratase activity and role in carbon utilization
AFUB_068370	Saccharopine dehydrogenase. Has domain(s) with predicted nucleotide binding, oxidoreductase activity and role in oxidation-reduction process
AFUB_072390	Putative integral plasma membrane heat shock protein
AFUB_072790	Regulator of G protein signaling domain protein (rgsA in nidulans)
AFUB_081960	Protein of unknown function
AFUB_084150	C-4 methyl sterol oxidase Erg25, putative
AFUB_089270	14-alpha sterol demethylase (Cyp51, Erg11)
AFUB_091500	Ortholog(s) have stearoyl-CoA 9-desaturase activity (SdeA)
AFUB_093140	Sterol delta 5,6-desaturase (Erg3)
AFUB_094880	Protein of unknown function
AFUB_096050	Ortholog(s) have IgE binding, thioredoxin peroxidase activity and extracellular region, peroxisome localization
AFUB_098170	C-4 methyl sterol oxidase, putative (Erg25)
AFUB_099590	Sterol Regulatory Element Binding Protein Family member (SrbB)

Supplementary table 8: Oleic acid (OA) supplementation rescues MBX-7591 mediated growth inhibition against *Aspergillus fumigatus* and *Cryptococcus neoformans*.

**Species/Strain**	**MIC OA**	**MIC MBX-7591**	**MIC OA combination**	**MIC MBX-7591 combination**	**FIC Value**
CEA10 *Aspergillus fumigatus*	> 500 ug/mL	2.45 µg/ml	7.81 µg/ml	9.81 µg/ml	4
CEA10 *Aspergillus fumigatus*	> 500 ug/mL	2.45 µg/ml	15.63 µg/ml	19.63 µg/ml	8
CEA10 *Aspergillus fumigatus*	> 500 ug/mL	2.45 µg/ml	31.25 µg/ml	> 19.63 µg/ml	> 8
H99 *Cryptococcus neoformans*	> 500 ug/mL	4.91 µg/ml	31.25 µg/ml	> 19.63 µg/ml	> 4

Supplementary Table 9: Stearoyl-CoA desaturase copy number in relevant fungal species.

Species	Strain ID	# of gene copies with both domains	Gene IDs
Rhizopus spp.	99-880	2	RO3G_00788<br>RO3G_09470<br>RO3G_16084
Cryptococcus neoformans	H99	1	CNAG_04687
P. variotii	N/A	N/A	
Apophysomyces spp.	N/A	N/A	
***Saksenaea* spp.**	N/A	N/A	
Blastomyces dermatitidis	ATCC 26199	1	BDFG_06311<br>BDFG_03868 / BDFG_03869
Blastomyces dermatitidis	ER-3	2	BDCG_06371<br>BDCG_01984
Coccidioides immitis	H538.4	1	CIHG_01912
Coccidioides immitis	RS	1	CIMG_08158
Coccidioides immitis	WA_211	1	DIZ76_014374
Coccidioides posadasii	C735 delta SOWgp	1	CPC735_021820
Coccidioides posadasii	RMSCC 3488	1	CPAG_05345
Coccidioides posadasii	Silveira 2022	1	CPSG_08402-t26_1
Histoplasma capsulatum	G184AR	1	I7I52_02739
Histoplasma capsulatum	G186AR	1	I7I50_11502
Histoplasma capsulatum	G217B	1	I7I48_07376

Histoplasma capsulatum	H143	1	HCDG_01170/ HCDG_01171<br>Two annotations next to each other but one gene
Histoplasma capsulatum	H88	1	I7I53_07531
Aspergillus fumigatus	Af293	1	Afu7g05920
Aspergillus fumigatus	A1163	1	AFUB_091500
Aspergillus flavus	NRRL3357 2020	4-6	F9C07_470<br>F9C07_1704 (B5 domain only 112 AA)<br>F9C07_2224608<br>F9C07_2283881<br>F9C07_4280<br>F9C07_7041
Aspergillus niger	CBS 513.88	2	An07g01960<br>An12g02690 (only 150 AA, B5 domain)<br>An12g09940
Aspergillus terreus	NIH2624	2	ATEG_06342<br>ATEG_09212
Aspergillus nidulans	FGSC A4	2	AN6731<br>AN4135
Candida albicans	SC5314	2	C1_08360C_A<br>C2_07090C_A
Mucor circinelloides	1006PhL	2	HMPREF1544_07845<br>HMPREF1544_11620
Mucor lusitanicus	CBS 277.49	2	QYA_156614<br>QYA_186878
Fusarium spp.		2-4 genes	
Scedosporium apiospermum	IHEM 14462	1	SAPIO_CDS7142
Candida auris	B8441	2	B9J08_002798<br>B9J08_003612
Saccharomyces cerevisiae	S288C	1	YGL055W

Supplementary Table 10. Strains used in this study

Species	Strain	Background strain	Genotype	Source
Aspergillus fumigatus	CEA10/CBS144.89	N/A	WT laboratory Strain	CBS
Aspergillus fumigatus	$_{P}$GpdA-Luciferase Reporter	CEA17	*pyrG1, gpdA(p) A. nidulans :: firefly luciferase ::trpc(T), pyrG+*	Cramer Laboratory, Geisel School of Medicine at Dartmouth [1]
Aspergillus fumigatus	RBH-AF-01	N/A	CF isolate	Darius Armstrong-James, Imperial College London
Aspergillus fumigatus	RBH-AF-02	N/A	CF isolate	Darius Armstrong-James, Imperial College London
Aspergillus fumigatus	RBH-AF-03	N/A	CF isolate	Darius Armstrong-James, Imperial College London
Aspergillus fumigatus	RBH-AF-08	N/A	CF isolate	Darius Armstrong-James, Imperial College London
Aspergillus fumigatus	RBH-AF-10	N/A	CF isolate	Darius Armstrong-James, Imperial College London
Aspergillus fumigatus	10-19-02-27	N/A	COPD isolate	Darius Armstrong-James, Imperial College London [2]
Aspergillus fumigatus	08-31-08-91	N/A	Left Ear, Otis externa	Darius Armstrong-James, Imperial College London [2]
Aspergillus fumigatus	08-19-02-10	N/A	Environmental Isolate	Darius Armstrong-James, Imperial College London [2]
Aspergillus fumigatus	08-19-02-46	N/A	Environmental Isolate	Darius Armstrong-James, Imperial College London [2]
Aspergillus fumigatus	08-19-02-61	N/A	Environmental Isolate	Darius Armstrong-James, Imperial College London [2]

Aspergillus fumigatus	F15390	N/A	CCPA with aspergilloma, then CFPA – clinical failure	Susan Howard, University of Manchester, UK [3]
Aspergillus fumigatus	F11628	N/A	CCPA with bilateral aspergillomas, CFPA -- clinical failure	Susan Howard, University of Manchester, UK [3]
Aspergillus fumigatus	AFS35	N/A	*Δaku70*	Scott Moye-Rowley, University of Iowa [4, 5]
Aspergillus fumigatus	*ΔatrR*	AFS35	*atrRΔ::hph*	Scott Moye-Rowley, University of Iowa [4, 5]
Aspergillus fumigatus	*ΔsrbA (SPF134)*	AFS35	*ΔsrbA::hph*	Scott Moye-Rowley, University of Iowa [4, 5]
Aspergillus fumigatus	*hspA-atrR (SPF108)*	AFS35	*hspA-atrR::ptrA*	Scott Moye-Rowley, University of Iowa [5]
Aspergillus fumigatus	*hspA-atrR, ΔsrbA (SPF137)*	AFS35	*hspA-atrR::ptrA, ΔsrbA::hph*	Scott Moye-Rowley, University of Iowa [5]
Aspergillus fumigatus	*ΔatrR, ΔsrbA*	*ΔsrbA (SPF134)*	*ΔsrbA::hph, ΔatrR::ptrA*	Cramer Laboratory, Geisel School of Medicine at Dartmouth
Aspergillus fumigatus	*ΔhapX*	CEA17	*ΔhapX::hph*	Hubertus Haas, Innsbruck Medical University [6]
	ΔhapX ΔsrbA	*ΔsrbA* (CEA17)	*ΔhapX::hph*	Hubertus Haas, Innsbruck Medical University [6]
Aspergillus fumigatus	ATCC46645	N/A	WT laboratory strain	ATCC
Aspergillus fumigatus	TDC19.77 GFP::SrbA	CEA17	pyrG-;srbA(p):GFP:SrbA:Ap pyrG	Cramer Laboratory, Geisel School of Medicine at Dartmouth
Aspergillus fumigatus	*Δcyp51A*	CEA17	*NiiA-cyp51A*	Terry Roemer, Merck [7]
Aspergillus fumigatus	*NiiA-cyp51A, Δcyp51B*	CEA17	erg11B::pyrG, *NiiA-cyp51A*	Terry Roemer, Merck [7]
Aspergillus fumigatus	*ΔsrbB*	CEA17	pyrG-;ΔsrbA:AppyrG	Cramer Laboratory, Geisel School of Medicine at Dartmouth

Aspergillus fumigatus	*Δerg25A*	CEA17	erg25A::*pyrG*	Cramer Laboratory, Geisel School of Medicine at Dartmouth [8]
Aspergillus fumigatus	*Δerg25B*	CEA17	*erg25B::pyrG*	Cramer Laboratory, Geisel School of Medicine at Dartmouth [8]
Aspergillus fumigatus	*Δerg5*	CEA17	ΔKu80	Cramer Laboratory, Geisel School of Medicine at Dartmouth
Aspergillus fumigatus	Xylose Inducible *sdeA*	CEA10	*pxylP:psdeA;hph*	Cramer Laboratory, Geisel School of Medicine at Dartmouth

SUPPLEMENTARY FIGURES:

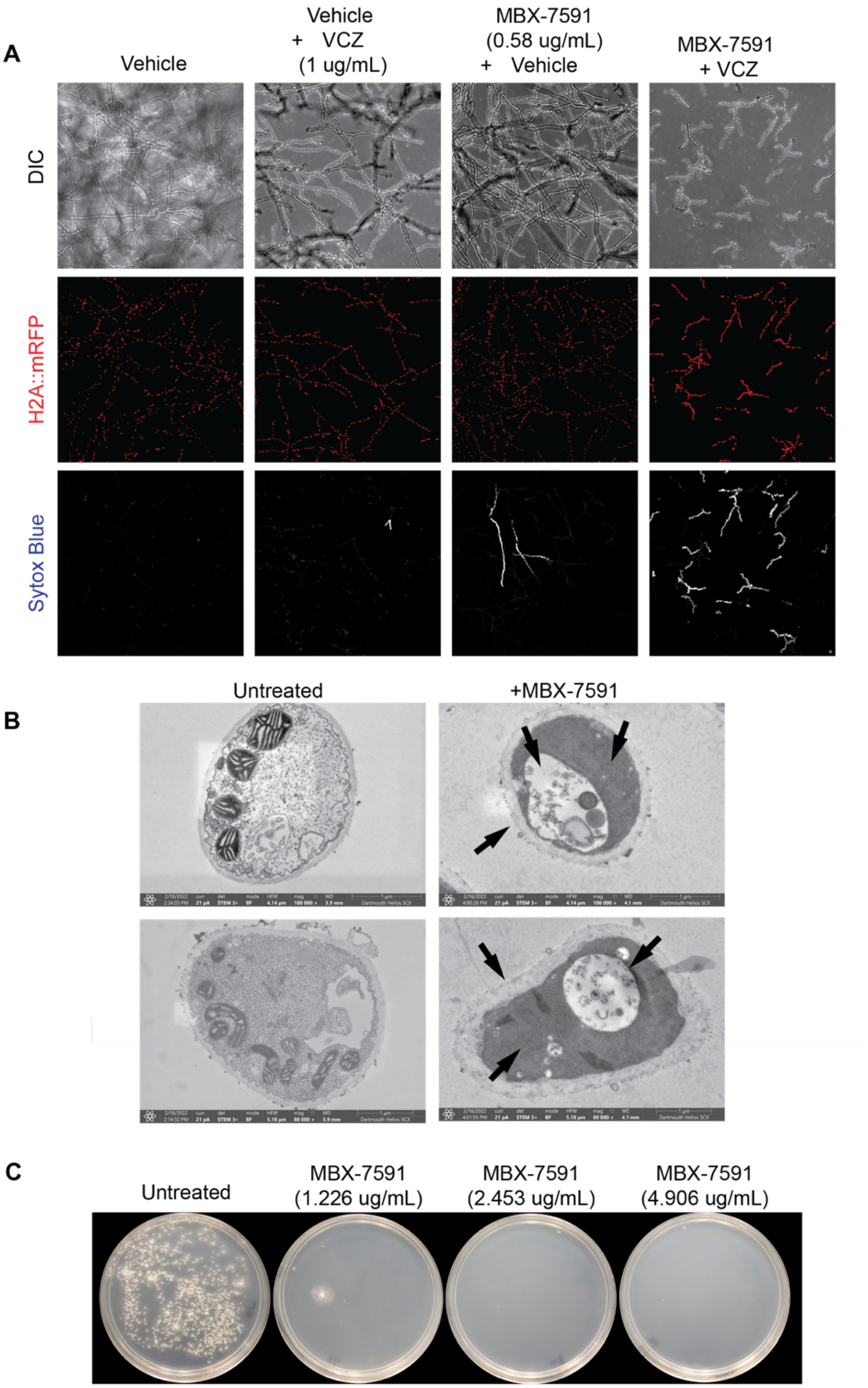

Supplementary Figure 1. MBX-7591 pre-treatment induces cell death.

A) Biofilms grown for 12 hours with MBX-7591 pre-treatment (PT) are more susceptible to subsequent 6-hour voriconazole (VCZ) treatment. MBX-7591 PT + VCZ biofilms show higher sytox staining and nuclear collapse indicative of increased cell membrane permeability and cell death. B) TEM image of cross section of *Aspergillus fumigatus* hyphae from 24-hour biofilms grown in vehicle control or 0.588 µg/ml MBX-7591. MBX-7591 induces changes in the cell wall, increased vacuolization and increased cytosolic density (black arrows). C) MBX-7591 reduces fungal colony recovery after treatment with half-MIC (1.226 µg/ml), MIC (2.453 µg/ml) and 2X MIC (4.906 µg/ml). CLSI assay done as described in methods and incubated for 48 hours at 37°C, 5%CO_2. Subsequently 100 µL of untreated control, ½ MIC, MIC and 2X MIC wells were plated onto GMM and incubated at 37°C, 5%CO_2 for 48h. Representative images of fungal growth of 2 independent biological replicates shown.

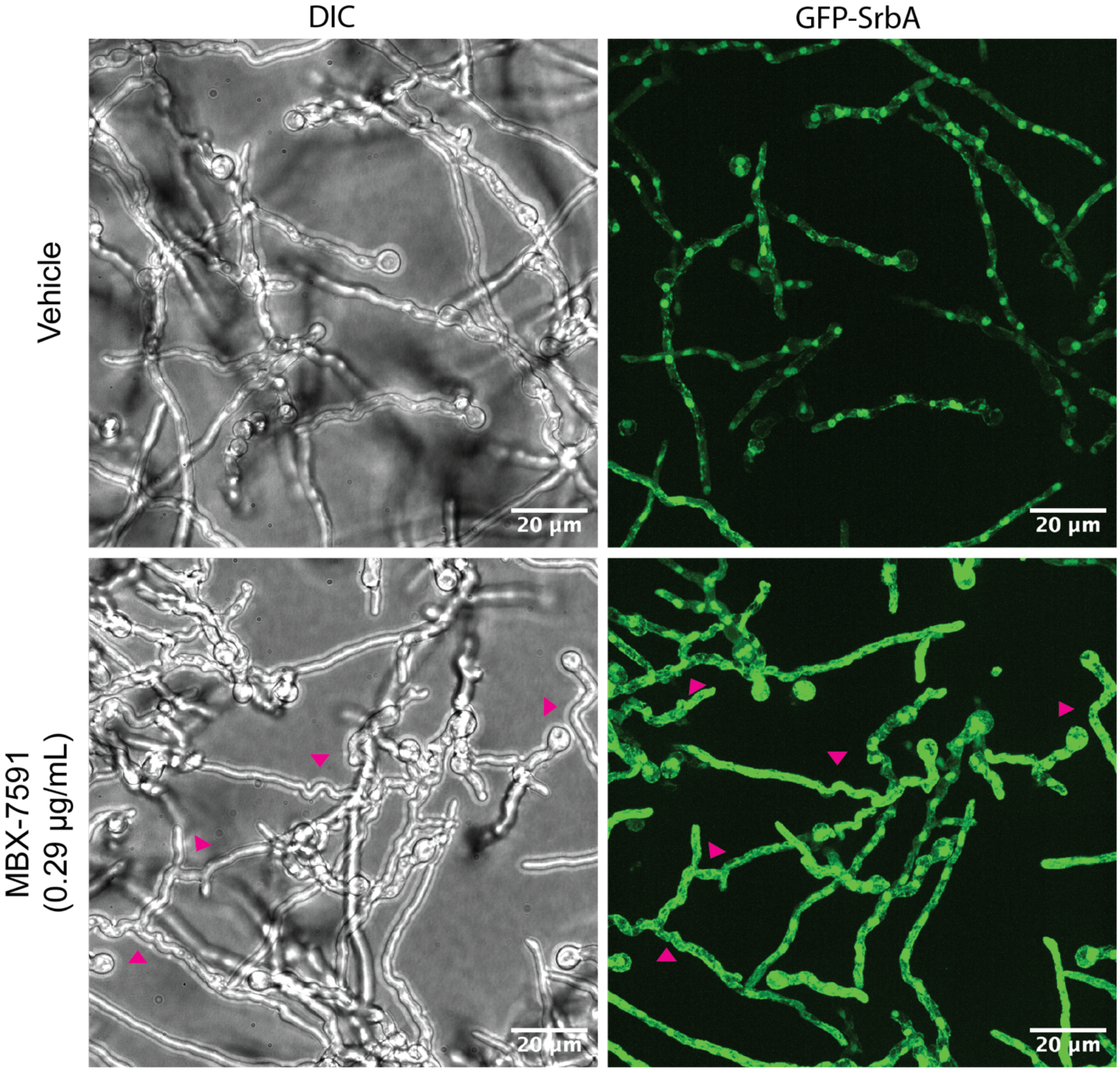

Supplementary Figure 2. Pre-treatment with MBX-7591 (0.29 µg/ml) induces morphological changes in 12-hour biofilms.

1x10^5 spores/mL CEA10 biofilms grown for 12 hours in liquid GMM with a sub-MIC MBX-7591 pre-treatment (PT , 0.29 µg/ml) show increased hyper-branching and crooked hyphae suggesting loss of polarity during hyphal growth due to MBX-7591 treatment compared to vehicle control (pink arrowheads).

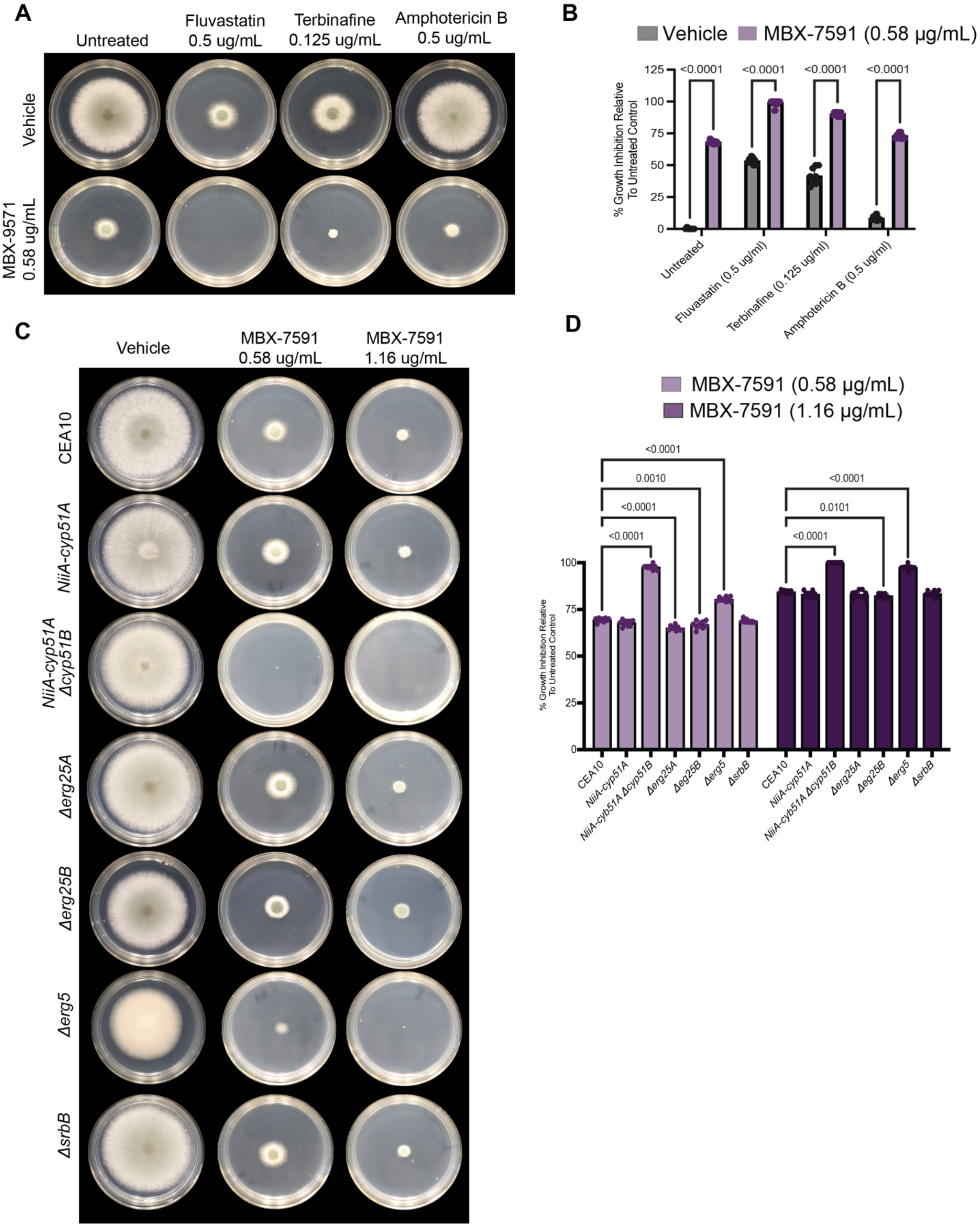

Supplementary Figure 3. MBX-7591 has synergy with ergosterol pathway inhibition.

A-B) colony biofilm assay shows MBX-7591 can potentiate fluvastatin (statin) and terbinafine (Allylamine) but not amphotericin B (Polyene). C-D) colony biofilm assay reveals specific null mutants in the ergosterol synthesis pathway have significant decreased susceptibility to MBX-7591.

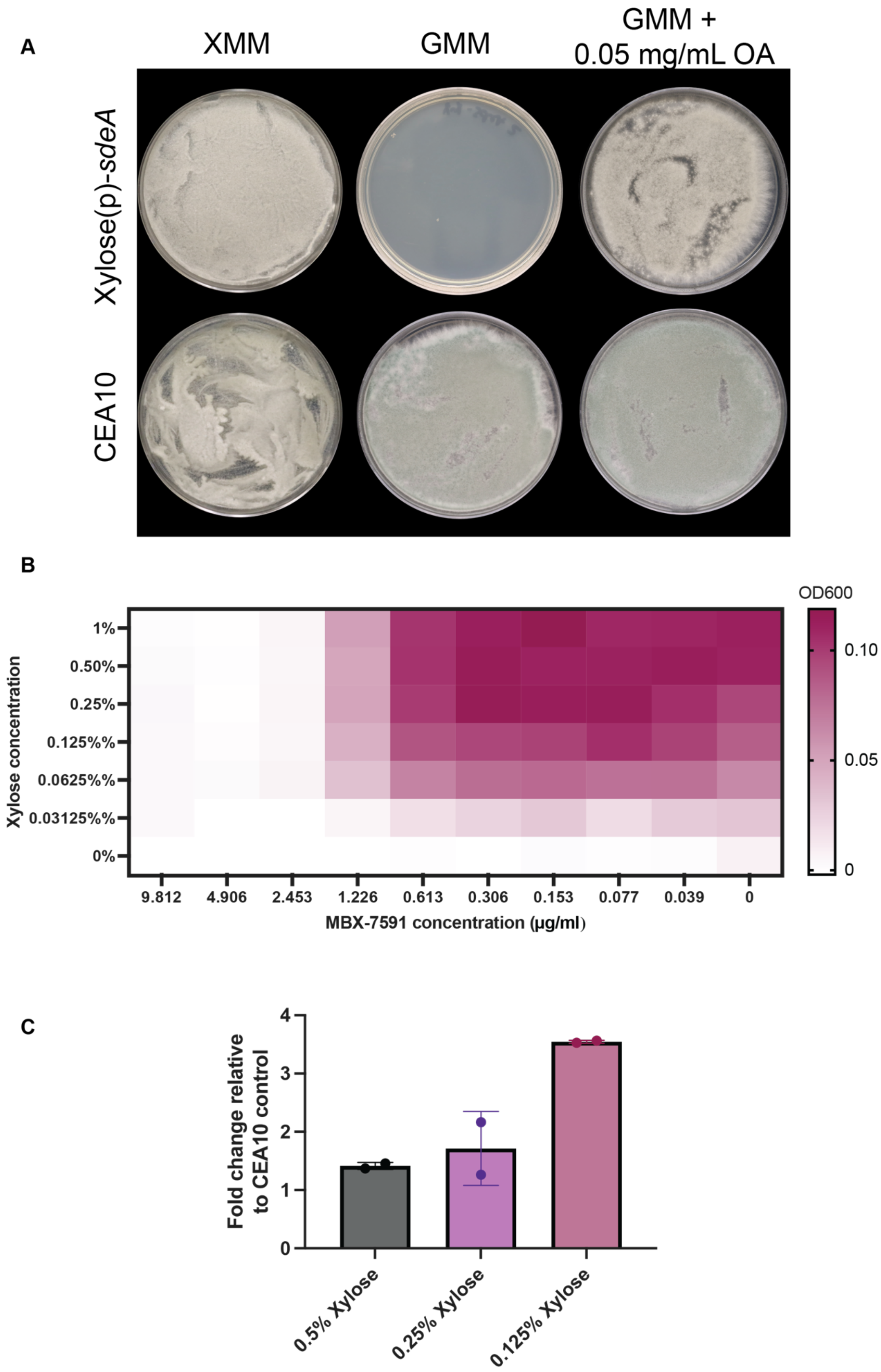
A
XMM
GMM
GMM +
0.05 mg/mL OA
Xylose(p)-sdeA
CEA10
B
OD600
0.10
0.05
0
Xylose concentration
1%
0.50%
0.25%
0.125%%
0.0625%%
0.03125%%
0%
9.812
4.906
2.453
1.226
0.613
0.306
0.153
0.077
0.039
0
MBX-7591 concentration (µg/ml)
C
Fold change relative
to CEA10 control
4
3
2
1
0
0.5% Xylose
0.25% Xylose
0.125% Xylose

Supplementary Figure 4. *Xylose(p)-sdeA* % xylose requires sdeA expression close to WT or OA supplementation to grow.

A) *Xylose(p)-sdeA* (xylose inducible sdeA) requires xylose or OA supplementation for growth.100 uL of $1x10^5$ spores/mL inoculated onto glucose minimal media (1% glucose), xylose minimal media (1% xylose) or glucose minimal media (1% glucose) + 0.05 mg/mL oleic acid. Plates incubated for 72h at 37°C, 5% CO_2. B) FIC checkerboard synergy assay with MBX-7591 and xylose shows that *xylose(p)-sdeA* strain susceptibility to MBX-7591 is not mediated by xylose concentration. Checkerboard plate done with different concentrations of xylose in the Y-axis (1% - 0%) and MBX-7591 concentrations in the X-axis (9.812 – 0 μg/ml). 100 uL of $5x10^4$ spores/mL inoculated in each well. Plates incubated for 48h at 37°C, 5% CO_2. OD600 measured and normalized to OD600 minus media only control. Heat map encompasses the mean of three independent biological replicates. C) *sdeA* expression with concentrations of xylose that allow for sufficient growth to extract RNA is at WT or higher levels in *xylose(p)-sdeA* strain. $1x10^5$ spores/mL of CEA10 and xylose(p)-sdeA grown for 24h in XMM (Xylose minimal media 0.5%, 0.25%, 0.125%) at 37°C, 5% CO_2. Biomass collected in TRIsure, RNA extracted, and cDNA synthetized as previously reported [9]. Cq values normalized to housekeeping gene beta tubulin. Values normalized to fold change relative to CEA10 grown in the equivalent xylose concentration.

Supplemental Methods:

Sytox cell death stain assessment of MBX-7591pre-treated and voriconazole treated biofilms:

To assess if MBX-7591 can sensitize biofilms to voriconazole, 100 μL of 1x10^5 spores/mL were inoculated into four wells of μ-Slide 8 Well Ibidi imaging plate. Two wells then received 100 μL of either media or 1.16 μg/ml MBX-7591 for a final concentration tested of 0.58 μg/ml. Ibidi plate was incubated at 37°C, 5% CO_2 for 12 hours to form a biofilm. At the 12-hour timepoint, voriconazole was added for a final concentration of 1 μg/ml to one MBX-7591 treated well, and one untreated well. Plate was then incubated for 6 more hours at 37°C, 5% CO_2. Biofilms were stained with Sytox blue for 5 minutes and imaged for GFP, RFP, DIC and Sytox stain.

TEM sample preparation:

1x10^7 spores/mL CEA10 grown for 24 hours in liquid GMM + vehicle control or 0.588 μg/ml MBX-7591 at 37°C, 5% CO_2 in a 15 mL conical. Samples briefly spun down at 5000 RPM for 10 minutes, and media removed. For transmission electron microscopy fungal biofilms were fixed as previously described [10]. Hyphal cross-sections were imaged using TALOS F200i.

Supplemental References:

1. Opperman, T.J., et al., *Luciferase-Based High-Throughput Screen with Aspergillus fumigatus to Identify Antifungal Small Molecules.* Methods Mol Biol, 2023. **2658**: p. 17-34.
2. Abdolrasouli, A., et al., *Genomic Context of Azole Resistance Mutations in Aspergillus fumigatus Determined Using Whole-Genome Sequencing.* mBio, 2015. **6**(3): p. e00536.
3. Howard, S.J., et al., *Frequency and evolution of Azole resistance in Aspergillus fumigatus associated with treatment failure.* Emerg Infect Dis, 2009. **15**(7): p. 1068-76.
4. Hagiwara, D., et al., *A Novel Zn2-Cys6 Transcription Factor AtrR Plays a Key Role in an Azole Resistance Mechanism of Aspergillus fumigatus by Co-regulating cyp51A and cdr1B Expressions.* PLoS Pathog, 2017. **13**(1): p. e1006096.
5. Paul, S., et al., *AtrR Is an Essential Determinant of Azole Resistance in Aspergillus fumigatus.* mBio, 2019. **10**(2).
6. Blatzer, M., et al., *SREBP coordinates iron and ergosterol homeostasis to mediate triazole drug and hypoxia responses in the human fungal pathogen Aspergillus fumigatus.* PLoS Genet, 2011. **7**(12): p. e1002374.

7. Hu, W., et al., *Essential gene identification and drug target prioritization in Aspergillus fumigatus.* PLoS Pathog, 2007. **3**(3): p. e24.
8. Blosser, S.J., et al., *Two C4-sterol methyl oxidases (Erg25) catalyse ergosterol intermediate demethylation and impact environmental stress adaptation in Aspergillus fumigatus.* Microbiology (Reading), 2014. **160**(Pt 11): p. 2492-2506.
9. Ross, B.S., et al., *Aspergillus fumigatus In-Host HOG Pathway Mutation for Cystic Fibrosis Lung Microenvironment Persistence.* mBio, 2021. **12**(4): p. e0215321.
10. Kowalski, C.H., et al., *Fungal biofilm morphology impacts hypoxia fitness and disease progression.* Nat Microbiol, 2019. **4**(12): p. 2430-2441.
